# Peripherally derived macrophages modulate microglial function to reduce inflammation after CNS injury

**DOI:** 10.1371/journal.pbio.2005264

**Published:** 2018-10-17

**Authors:** Andrew D. Greenhalgh, Juan G. Zarruk, Luke M. Healy, Sam J. Baskar Jesudasan, Priya Jhelum, Christopher K. Salmon, Albert Formanek, Matthew V. Russo, Jack P. Antel, Dorian B. McGavern, Barry W. McColl, Samuel David

**Affiliations:** 1 Centre for Research in Neuroscience, The Research Institute of the McGill University Health Center, Quebec, Canada; 2 Laboratory of Nutrition and Integrated Neurobiology, UMR INRA 1286, University of Bordeaux, Bordeaux, France; 3 Neuroimmunology Unit, Montreal Neurological Institute, McGill University, Montreal, Quebec, Canada; 4 Viral Immunology and Intravital Imaging Section, National Institute of Neurological Disorders and Stroke, National Institutes of Health, Bethesda, Maryland, United States of America; 5 UK Dementia Research Institute, University of Edinburgh, Edinburgh, United Kingdom; UCSD, United States of America

## Abstract

Infiltrating monocyte-derived macrophages (MDMs) and resident microglia dominate central nervous system (CNS) injury sites. Differential roles for these cell populations after injury are beginning to be uncovered. Here, we show evidence that MDMs and microglia directly communicate with one another and differentially modulate each other’s functions. Importantly, microglia-mediated phagocytosis and inflammation are suppressed by infiltrating macrophages. In the context of spinal cord injury (SCI), preventing such communication increases microglial activation and worsens functional recovery. We suggest that macrophages entering the CNS provide a regulatory mechanism that controls acute and long-term microglia-mediated inflammation, which may drive damage in a variety of CNS conditions.

## Author summary

The immune and the central nervous systems are now thought to be inextricably linked. In response to injury, the immune system shapes CNS recovery through a complex of molecular and cellular mediators. However, it is unclear how the kinetics, magnitude, and components of this response can be harnessed to improve CNS restoration. The two immune cells that dominate CNS lesions are resident microglia—already present before the injury—and infiltrating macrophages, which enter from the blood after injury. Both cells are thought to be critical to the outcome, yet it is unknown if, or how, they interact. To investigate this, we used mouse and human cells in microglia–macrophage coculture systems and an in vivo model of traumatic spinal cord injury. We show that infiltrating macrophages suppress key functions of microglia, such as removal of tissue debris and propagation of inflammation. Preventing macrophage–microglia communication increases microglial activation and worsens recovery. We suggest that infiltrating macrophages from the blood provide a natural control mechanism against detrimental acute and long-term microglial-mediated inflammation. Manipulation of the peripheral macrophages may provide a therapeutic treatment option to target microglial-mediated mechanisms that cause or exacerbate CNS injury and disease.

## Introduction

The immune system plays a pivotal role in development and homeostatic functions of the central nervous system (CNS) [1]. Immune system dysfunction can give rise to CNS disease [2] and its response to injury shapes recovery [3–5]. The cellular response to CNS injuries is stereotyped and involves the rapid reaction of tissue-resident microglia [6, 7] and the recruitment of myeloid cells such as neutrophils and monocyte-derived macrophages (MDMs) within days [5], and the activation of lymphocytes from the blood, meninges, and choroid plexus [8]. Two cell types that dominate CNS lesions are resident microglia and infiltrating MDMs. It is known that microglia and MDMs are ontogenetically distinct [9, 10], express cell type–specific transcripts and proteins [11, 12], and can thus potentially perform different functions at the site of injury [13–16]. However, their relative contribution to the injury response, and subsequent recovery, remains unclear. It is not known if these two cell types interact to specifically modulate each other’s function.

Two of the primary functions of microglia and MDMs during CNS injury are phagocytosis and propagation of inflammation [17]. We have shown previously that after traumatic spinal cord injury (SCI), cessation of microglia phagocytosis coincides with the infiltration of MDMs [14]. In animal models of stroke and CNS autoimmune disease, expression profiling of microglia after injury or at the onset of disease (at the point of MDM infiltration) shows that pathways involving core functions of microglia, such as inflammation, RNA transcription, and phagocytosis, are significantly down-regulated [16, 18, 19]. We therefore hypothesized that MDMs entering the CNS signal to resident microglia and modulate their function.

## Results

### Increasing numbers of infiltrating macrophages at CNS lesion site correlates with reduction in microglial phagocytosis

**Fig 1:**
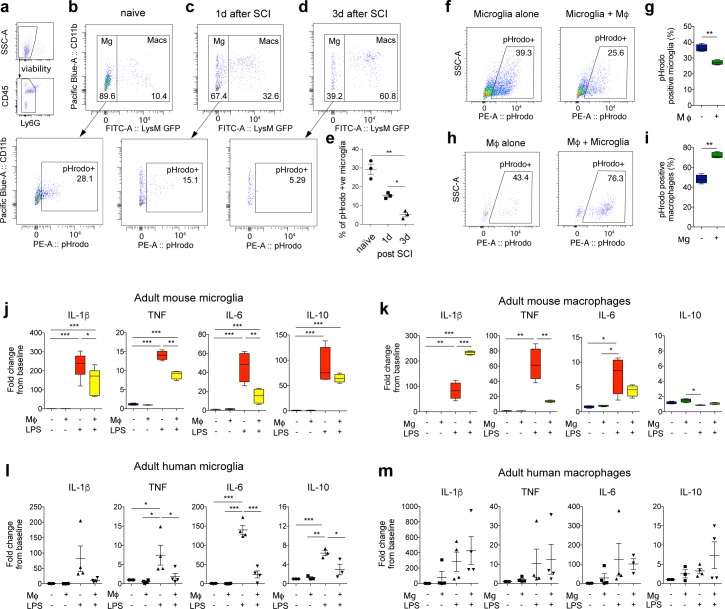
Reciprocal signaling between macrophages and microglia produces divergent functions in each cell type. (**a-e**) Increasing numbers of MDMs in the injured spinal cord correlates with reduced phagocytosis by microglia. Myeloid cells were isolated from the uninjured or injured spinal cord of LysM-eGFP mice and immediately treated with pHrodo- labeled myelin for four hours. (**a**) Flow cytometry gates to isolate microglia/macrophage populations exclude Ly6G+ neutrophils. (**b-d**) Representative flow cytometry dot plots show proportions of CD11b^+^/LysM-eGFP^−ve^ (microglia) and CD11b^+^/LysM-eGFP^+ve^ (macrophages) in uninjured or SCI tissue, 1 and 3 d after injury. Bottom panels show representative plots of microglia positive for pHrodo-myelin. (**e**) Graph shows reduced phagocytosis by microglia, with increasing presence of MDMs post-SCI. (**f-i**) In vitro bilaminar coculture of adult mouse microglia and macrophages. Representative FACS plots show uptake of pHrodo-labeled myelin in microglia (**f**) or macrophages (Mϕ) (**h**) alone (left panels) or cells in coculture (right). Graphs showing that phagocytosis is significantly decreased in microglia in the presence of Mϕ compared to microglia alone (**g**), and significantly increased in macrophages in the presence of microglia (**i**). (**j-m**) The bilaminar culture system was used to assess microglia–macrophage communication on inflammatory gene expression in adult mouse and human cells. (**j**) Adult mouse microglial gene expression of four key inflammatory cytokines (IL-1β, TNF, IL-6, and IL-10) treated with LPS (100 ng/mL) in the presence or absence of macrophages (Mϕ). (**k**) Adult mouse macrophage gene expression in the presence or absence of adult mouse microglia. **(l**) Adult human microglial gene expression in the presence or absence of human macrophages. (**m**) Adult human macrophage gene expression in the presence or absence of adult human microglia. Statistical analysis for (**e**), one-way ANOVA with Bonferroni corrections (*n =* 3); (**g, i**) Student *t* test (*n =* 4). (**j-m**) Two-way ANOVA with Bonferroni corrections (*n =* 3–6), mean ± SEM, *p < 0.05; **p < 0.01; ***p < 0.001. Corresponding raw data (**S1 Data**). FACS, fluorescently activated cell sorting; IL, interleukin; LPS, lipopolysaccharide; MDM, monocyte-derived macrophage; SCI, spinal cord injury; TNF, Tumor necrosis factor.

Three to five days after CNS injury, infiltrating macrophages are distributed across lesion sites and are therefore potentially able to interact with microglia [14, 19, 20]. After SCI, resident microglia and MDMs both increase in number around the lesion site [21]. We observed that MDMs and microglia are often in close proximity to one another (**S1A Fig**); however, whether they can regulate each other’s functions is not known. To assess whether the cessation of microglial phagocytosis seen after SCI correlates with the entry of MDMs into the CNS, as reported previously [14], we used antibody-conjugated magnetic-bead sorting to isolate CD11b^+^ cells from spinal cord lesions in LysM-eGFP knock-in mice. LysM-eGFP reporter mice strongly express eGFP in myelomonocytic cells [22] but in less than 3% of microglia after SCI and other CNS injuries [19, 20, 23]. Therefore, isolated CD11b^+^ positive cells could be defined as resident microglia (CD11b^+^/eGFP^−ve^) or infiltrating MDMs (CD11b^+^/eGFP^+ve^/Ly6G^−ve^) after extraction from the injured spinal cord. To assess the effect of infiltrating cells on resident microglia, cells were isolated from spinal cord lesions prior to significant MDM infiltration (one day) and after infiltration (three days). Immediately after isolation, cells were placed in vitro and incubated with pHrodo-labeled myelin for four hours. Flow cytometric analysis revealed that increasing numbers of MDMs at the lesion site correlates with a significant reduction in microglial phagocytosis (**Fig 1A–1E**). We therefore hypothesized that MDMs entering the CNS signal to resident microglia and modulate their function. As an additional note, we detected significantly more MDMs one day after injury, as compared to naïve spinal cord (**S1B Fig**). This is likely to represent macrophages in the process of entering the tissue, either through the vasculature or the meninges but before they have been reported to be seen in the parenchyma. It is possible that they could release signaling molecules from these locations that influence microglia, as seen in the reduction of phagocytosis at day one after SCI, compared to uninjured (naïve) mice (**Fig 1E**). There is a further significant increase in infiltration of MDMs by day three, compared to one day after SCI (**S1B Fig**).

### Development of an in vitro system to assess macrophage–microglial interactions

To directly test the hypothesis that MDMs modulate microglial function, we created a bilaminar culture system by plating bone marrow–derived macrophages (BMDMs) on coverslips with small paraffin spacers, which were then placed into wells containing adult microglia (**S2D Fig**). Primary adult mouse microglia were cultured under conditions that retain a transcriptional profile more similar to their in vivo counterparts, as compared to other media conditions, primary microglial cultures from neonates or microglial cell lines [11] (**S2A and S2B Fig)**. Other features, such as genes that reflect region-specific factors or function, may be altered when cells are placed in culture. The addition of Transforming growth factor (TGF)-β was not only necessary for a gene expression profile more similar to freshly isolated microglia but also showed greater ramified morphology in culture at seven days (**S2C Fig)**. Microglia “signature” genes were down-regulated during lipopolysaccharide (LPS)-induced inflammation, which supports their description as homeostatic [11]. There is no further modulation of these microglial genes in the presence of macrophages (**S2E Fig)**. As a control for cell numbers, we assessed modulation of these microglial genes by coculturing with microglia instead of BMDMs (**S2F Fig**). Suppression of inflammatory genes in adult microglia does not occur when cocultured with adult microglia. For these experiments, adult mouse microglia were cultured with or without adult microglia and stimulated with LPS (100 ng/mL) (**S2F Fig**).

### Macrophages suppress microglial phagocytic activity, whereas microglia enhance phagocytosis by macrophages

Using this bilaminar in vitro system, we assessed if soluble factors released by these two cell types affect phagocytic function in one another. Microglia and BMDMs were cocultured in the bilaminar system for 24 hours, separated, and incubated with pHrodo-labeled myelin for 90 minutes. Phagocytic uptake was assessed with flow cytometry. Uptake of pHrodo-labeled myelin was significantly decreased in microglia cultured in the presence of BMDMs compared with microglia cultured alone (**Fig 1F and 1G**). Surprisingly, myelin phagocytosis by BMDMs was significantly increased after coculture with adult microglia (**Fig 1H and 1I**). These findings reveal direct communication between the two cell types divergently affecting phagocytic function.

### Macrophages directly suppress inflammatory gene expression in adult mouse and human microglia

We next investigated macrophage effects on inflammatory gene expression in microglia and macrophages from adult mice and humans. We recently described a mathematical model of cytokine signaling, which found four inflammatory cytokines, interleukin (IL)-1β, tumor necrosis factor (TNF), IL-6, and IL-10, to be key nodes in the inflammatory network [24]. After LPS stimulation, coculture with BMDMs significantly down-regulated these genes in mouse and human microglia (**Fig 1J and 1L**). In contrast, IL-1β expression was increased in LPS-stimulated macrophages cocultured with mouse microglia (**Fig 1K**). There were nonsignificant trends towards increases in inflammatory cytokines in human macrophages in the combined presence of LPS and microglial cells (**Fig 1M**), contrasting with the significant suppression of these genes in microglia in the same conditions (**Fig 1L**). These experiments show the direct suppressive effects of macrophages on microglia, and reciprocal but divergent effects of microglia on macrophages.

**Fig 2:**
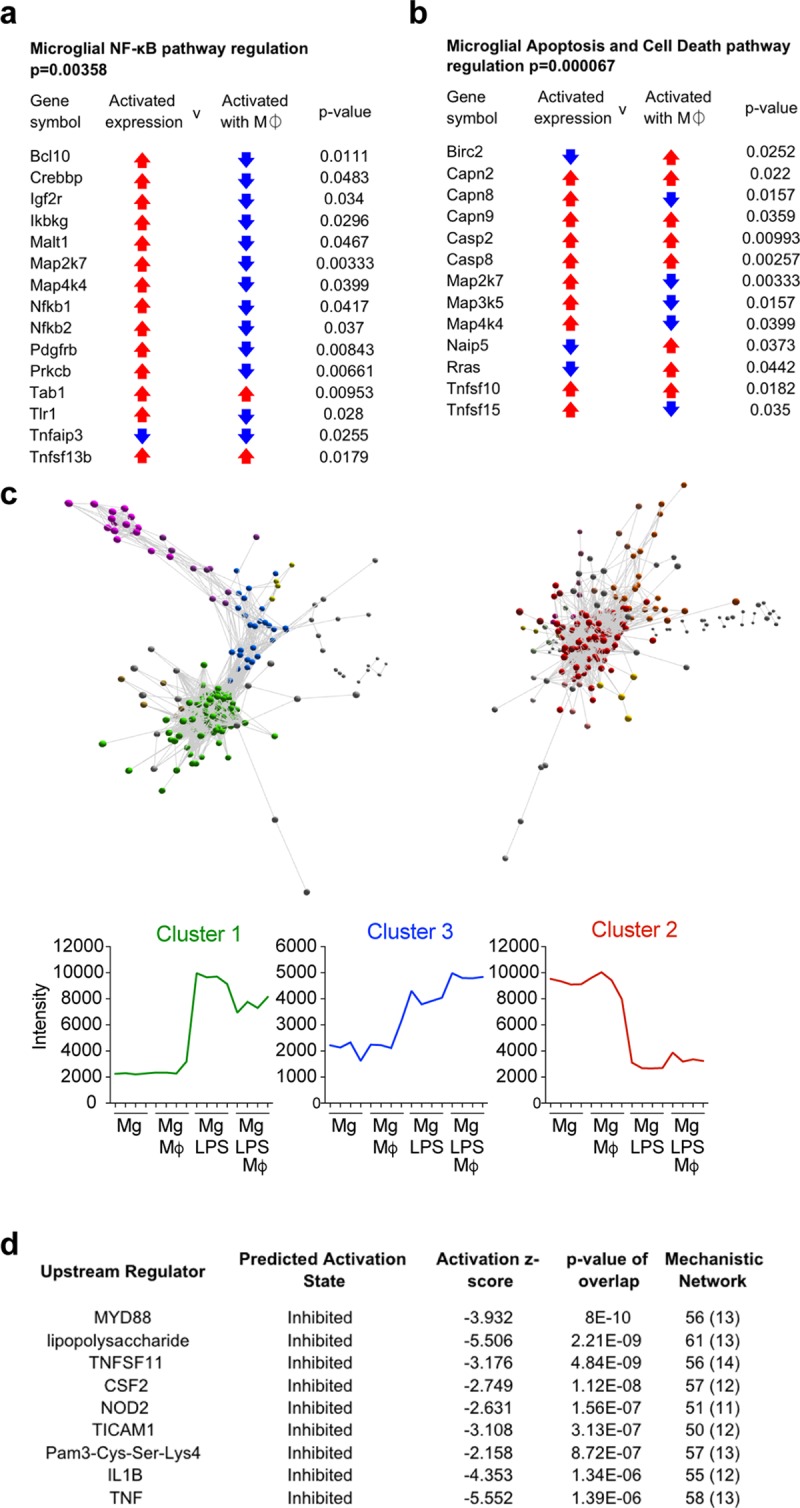
Transcriptional profiling reveals macrophages suppress key inflammatory pathways and dysregulate apoptotic cell death pathways in adult mouse microglia. Transcriptional profiling of mouse microglia revealed 1,076 differentially expressed genes between LPS-treated microglia in the presence or absence of macrophages. (**a, b**) IPA of the top dysregulated pathways within differentially expressed transcripts. NF-κB (**a**) and apoptosis and cell death (**b**) were the most significantly dysregulated in terms of number of genes and significance. Arrows in the “Activated expression” column indicate individual gene regulation when the pathway is induced in LPS-stimulated microglia (red = up-regulated, blue = down-regulated). Arrows in “Activated with Mϕ” column indicate individual gene regulation of LPS-stimulated microglia in the presence of macrophages. (**c**) A transcript-to-transcript correlation network plot of transcripts significantly changed in LPS-stimulated microglia in the presence of macrophages; network plot generated with Miru software (Pearson correlation threshold, *r* ≥ 0.86). Nodes in panel **c** represent transcripts (probe sets), and edges (connecting lines) represent the degree of correlation in expression between them. The network plot was clustered using a Markov clustering algorithm, and transcripts were assigned a color according to cluster membership. Graphs shown below are the mean expression profile of all transcripts within clusters 1, 2, and 3 (*n =* 4 per group). (**d**) Table shows upstream regulators of transcripts identified as differentially regulated between LPS-treated microglia and LPS-treated microglia in the presence of macrophages. The second column defines the predicted activation state of the upstream regulator when macrophages are present. The numbers in the last column indicate the total number of molecules in the data set that are downstream of the regulators, and the numbers in parenthesis indicate the total number of regulators involved in that particular network. Corresponding raw data (**S1 Data**). IL, interleukin; IPA, ingenuity pathway analysis; LPS, lipopolysaccharide; Mϕ, macrophage.

In the bilaminar culture system in mouse and human microglia, we found significant suppression of pro-inflammatory cytokines IL-1β, TNF, and IL-6 in the presence of macrophages (**Fig 1J and 1L**). We also found a significant reduction in human microglia of IL-10, a canonical brake on inflammation [25, 26]. Despite IL-10’s opposing function to the pro-inflammatory cytokines, all four cytokines are increased with LPS 24 hours after stimulation. We have recently described the complexities of cytokine networks over time and shown how the modulation of one cytokine in the system may result in varying temporal kinetics of the others [26]. Here, we examined a single time point, but as all four cytokines were suppressed by the presence of macrophages, we sought to examine whether microglial transcription, in general, became globally suppressed by macrophages. To understand the global effects of macrophage suppression on microglia, we transcriptionally profiled LPS-activated adult mouse microglia in the presence or absence of macrophages. A total of 1,076 genes were significantly differentially regulated in activated microglia in presence of macrophages, with approximately 50% up-regulated and 50% down-regulated. Ingenuity pathway analysis (IPA) revealed that the most dysregulated canonical signaling pathways were those related to nuclear factor (NF)-κB signaling, a master regulator of inflammation (**Fig 2A**) and apoptosis and cell death (**Fig 2B**). Network analysis revealed three major clusters of genes distributed across two distinct regions reflecting distinct gene co-expression patterns (**Fig 2C**). In the major gene cluster 1 (185 genes), which comprised genes down-regulated within microglia in the presence of macrophages, we found that the top upstream regulators, predicted to be inhibited with high confidence, included MyD88, IL-1β, and TNF (**Fig 2D**). These analyses support our findings that gene expression within the major inflammatory cascades in microglia is suppressed in the presence of macrophages.

### Prostaglandin E_2_ signaling via the EP2 receptor regulates macrophage-mediated suppression of microglial function

**Fig 3:**
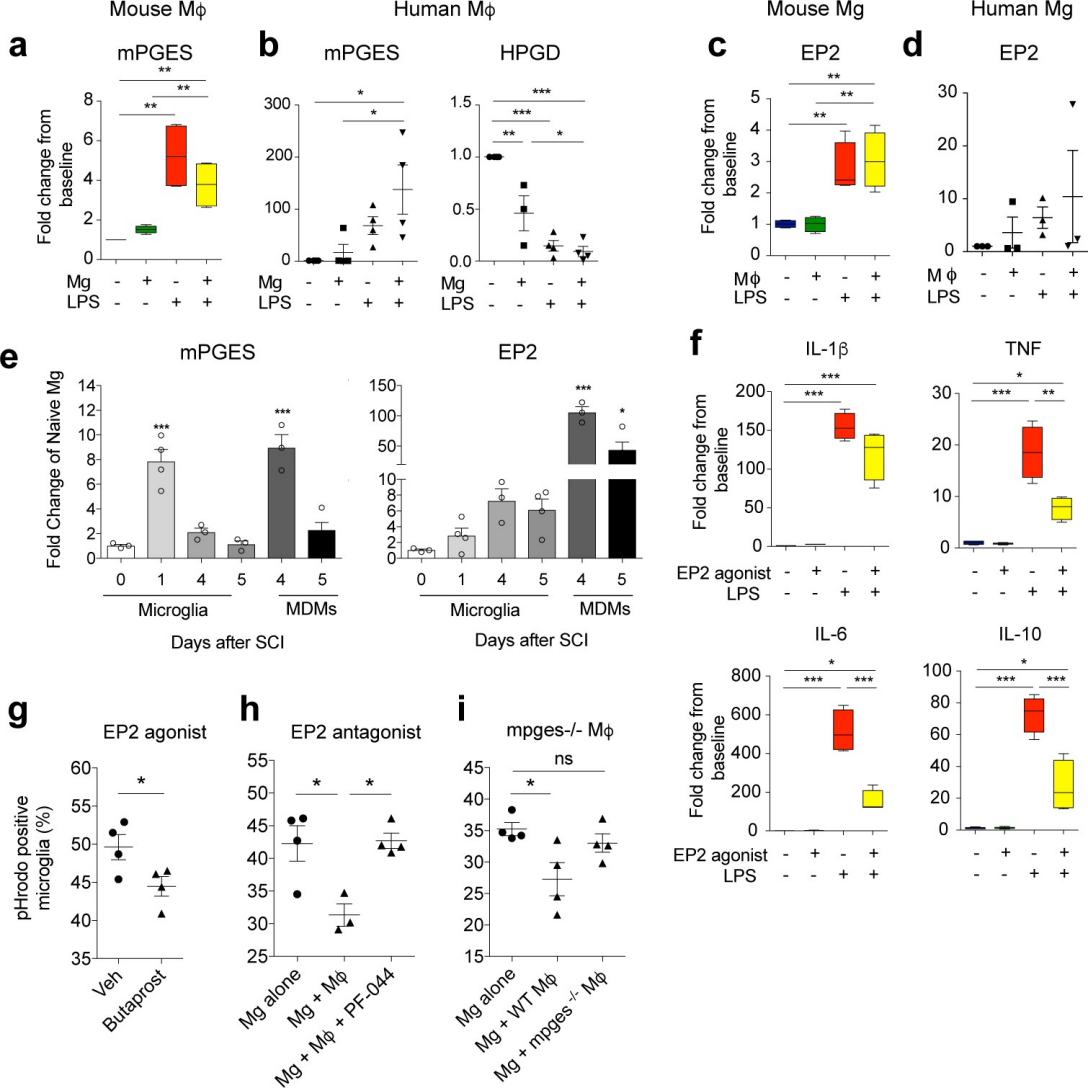
PGE_2_ signaling via the EP2 receptor is responsible for macrophage-mediated suppression of microglia. (**a-d**) The bilaminar culture system was used to assess expression of inducible mPGES and EP2 receptor in adult mouse and human microglia and HPGD expression in human macrophages. (**e**) mPGES and EP2 receptor mRNA expression in FAC-sorted cells 1–5 days after SCI in mice. (**f**) Adult mouse microglial mRNA expression of four key inflammatory cytokines (IL-1β, TNF, IL-6, and IL-10) treated with LPS (100 ng/mL) in the presence or absence of EP2 agonist, Butaprost (1 μM). (**g**) Phagocytosis of pHrodo-labeled myelin by adult mouse microglia is reduced by pretreatment with Butaprost (1 μM; one hour). (**h**) Phagocytosis of pHrodo-labeled myelin by adult mouse microglia, either alone or in the presence of macrophages (Mϕ) treated with vehicle or EP2 receptor antagonist, PF-0441894 (10 μM). (**i**) Phagocytosis of pHrodo-labeled myelin by adult mouse microglia alone or in the presence of WT macrophages or macrophages from *mPGES*^*−/−*^ mice. Statistical analysis for (**a-d, f**), two-way ANOVA with Bonferroni corrections (*n =* 3–4), and (**e, g-i**), one-way ANOVA with Bonferroni corrections (*n =* 3–4). Mean ± SEM. *p < 0.05; **p < 0.01; ***p < 0.001. Corresponding raw data (**S1 Data**). FAC, fluorescently activated cell; HPGD, hydroxyprostaglandin dehydrogenase; IL, interleukin; LPS, lipopolysaccharide; mPGES, microsomal prostaglandin E synthase-1; PGE_2_, prostaglandin E_2_; SCI, spinal cord injury; TNF, tumor necrosis factor; WT, wild-type.

As IL-1β was one of the key cytokines to be differentially regulated in microglia and macrophages in the coculture experiments (**Fig 1J and 1K**), we searched for factors known to regulate IL-1β in microglia. Prostaglandin E_2_ (PGE_2_) signaling via the EP2 receptor has been reported to reduce IL-1β expression in microglia [27]. In addition, EP2 receptor signaling has also been reported to reduce phagocytosis [28–30]. We therefore hypothesized that PGE_2_ signaling via microglial EP2 receptors could be responsible for the suppressive effects of macrophages. Inducible microsomal prostaglandin E synthase-1 (mPGES) and EP2 receptor were up-regulated during inflammation in mouse and human microglia and macrophages in vitro (**Fig 3A–3D** and **S3A Fig**) and in vivo in mice after SCI (**Fig 3E**). EP2 receptor expression was also up-regulated 22-fold when assessed by transcriptional array (**S3A Fig**). Transcript levels for EP1 and 4 were down-regulated in mouse microglia in vitro, suggesting they are not involved (**S3A Fig**). mPGES and hydroxyprostaglandin dehydrogenase (HPGD) work in concert to regulate PGE_2_ production and release as HPGD converts PGE_2_ to its biologically inactive metabolite [31]. In human macrophages, mPGES was significantly increased and HPGD was significantly down-regulated during inflammation (LPS) when cocultured with microglia, compared with macrophages cultured alone (without LPS) (**Fig 3B**), suggesting greater PGE_2_ production in stimulated macrophages in the presence of microglia. The expression of EP2 receptor in human microglia was not significantly up-regulated upon stimulation with LPS (**Fig 3D**). However, the data show that it is expressed, allowing the cells to detect PGE_2_.

Taken together, these experiments show that the components needed to allow PGE_2_ signaling at the EP2 receptor in microglia are up-regulated during inflammation and may be utilized for macrophage–microglia communication.

To functionally assess the role of the EP2 receptor, we treated adult mouse microglia with the EP2 specific agonist, Butaprost. Treatment of microglia significantly reduced TNF, IL-6, and IL-10 in the same manner as the macrophage-mediated suppression of these genes (**Fig 3F**). This contrasted with the effects of Butaprost on BMDMs, which only reduced TNF expression (**S3E Fig**). Butaprost also significantly reduced phagocytosis by microglia (**Fig 3G**). Macrophage suppression of microglial phagocytosis was rescued by the selective EP2 antagonist, PF-04418948 [32] (**Fig 3H**). In addition, unlike wild-type (WT) BMDMs, macrophages that lack mPGES (*mpges −/−*) do not suppress microglial phagocytosis (**Fig 3I**). Taken together, these results show that PGE_2_ produced by peripherally derived macrophages plays a major role in suppression of microglial phagocytic function via EP2 receptors.

**Fig 4:**
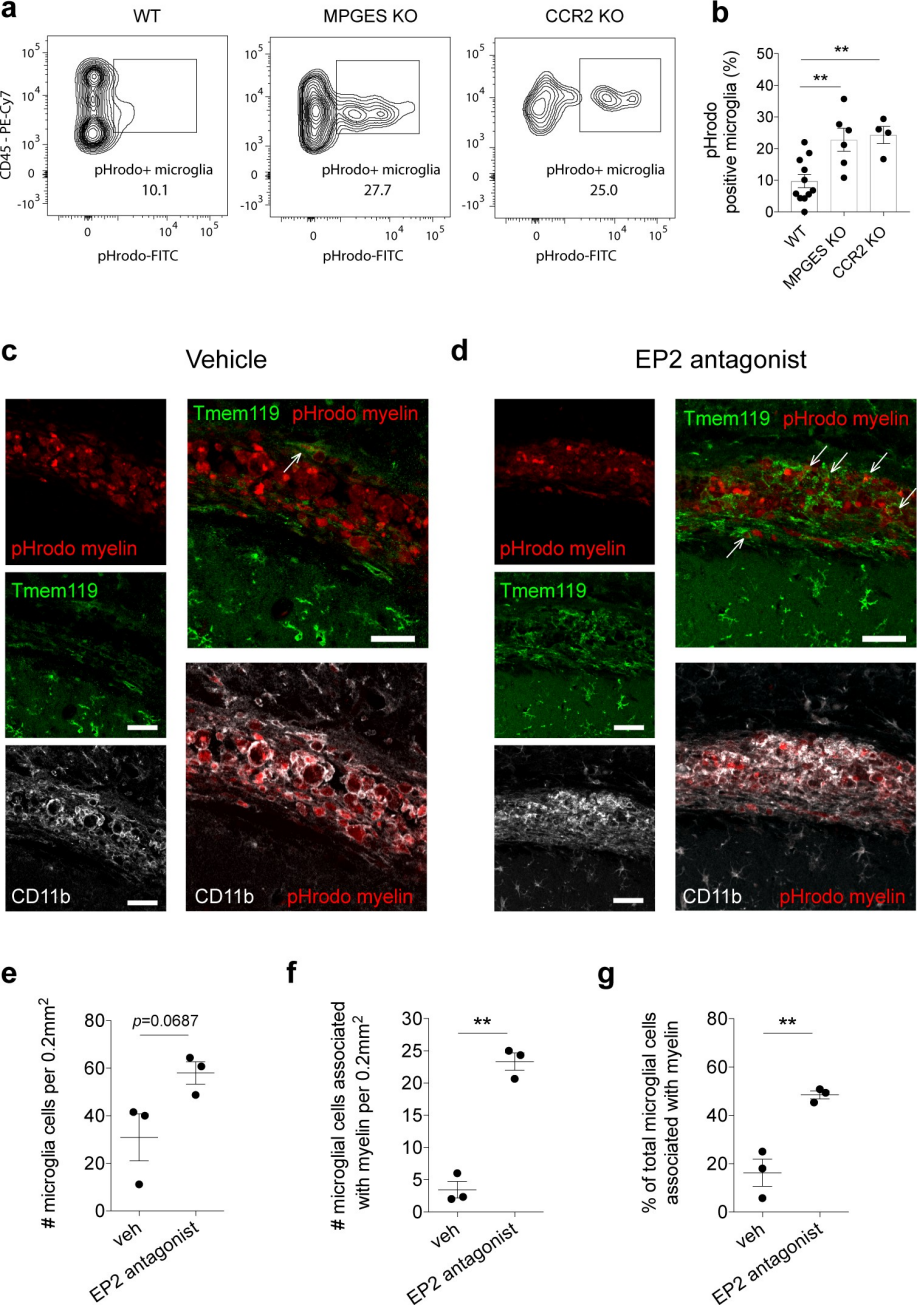
Lack of macrophages and loss of PGE_2_ signaling via the EP2 receptor increases microglia phagocytosis in vivo. (**a-b)** Myeloid cells were isolated from the injured spinal cord of WT, *mpges*^*−/−*^, and CCR2 KO mice three days after SCI, and immediately treated with pHrodo (green)-labeled myelin for four hours. MDM and neutrophil populations were excluded by gating CD11b^+^/CD45^+^/Ly6G^−ve^/Ly6C^−ve^ cells. (**a**) Representative FACS plots show proportions of microglia positive for pHrodo (Green)-myelin. (**b**) Graph shows significantly increased phagocytosis by microglia after SCI in *mpges*^*−/−*^ and CCR2 KO mice compared with WT control. (**c-g**) In vivo injection into the corpus callosum of pHrodo-labeled myelin in conjunction with vehicle (control) or EP2 receptor antagonist (PF-0441894; 1 μM) in WT mice. Association of microglia (Tmem119; green) with pHrodo-myelin (red) was assessed at the site of injection in the two groups, vehicle (**c**) and EP2 receptor antagonist (**d**). In the EP2 antagonist–treated group, there is an increase in the number of microglia at the injection site (**e**), the number of microglia that contact or contain pHrodo-myelin (**f**), and the percentage of total microglia that are in contact or contain pHrodo-myelin (**g**), compared with controls. Arrows indicate transmembrane protein (Tmem)119+ microglia containing or in contact with pHrodo-myelin. Statistical analysis for (**b**), one-way ANOVA with Bonferroni corrections (*n =* 4–10), and (**e-g**) Student *t* test (*n =* 3). Mean ± SEM. **p < 0.01; scale bars = 50 μm. Corresponding raw data (**S1 Data**). CCR2, C–C chemokine receptor type 2; FACS, fluorescently activated cell sorting; KO, knock-out; MDM, monocyte-derived macrophage; *mpges*, microsomal prostaglandin E synthase-1; PGE_2_, prostaglandin E_2_; SCI, spinal cord injury; Tmem, transmembrane protein; veh, vehicle; WT, wild-type.

**Fig 5:**
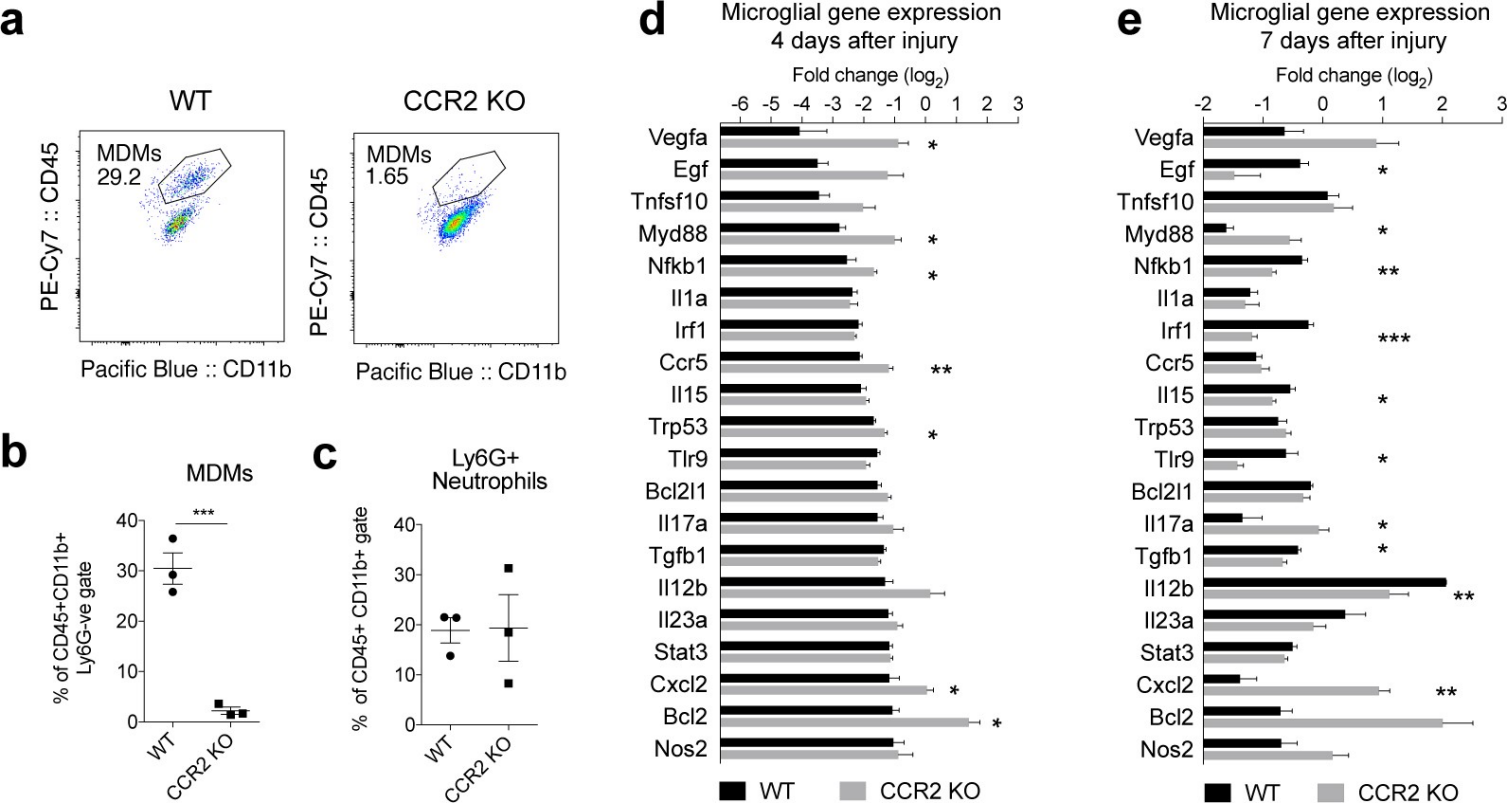
Lack of macrophage infiltration after SCI dysregulates microglial inflammation. Spinal cord contusion injury was performed in *CCR2*^*(rfp/rfp)*^ (CCR2 KO) mice and WT controls. (**a**) FACS plots show Ly6G^−ve^/CD45^hi^/CD11b^+^ MDMs in WT versus CCR2 KO mice five days after SCI. (**b**) Graph shows marked reduction in MDMs in the spinal cord lesion five days after SCI. (**c**) No change in Ly6G+ neutrophils in the injured cord at this time point (five days) post-SCI. (**d-e**) RT-qPCR gene array analysis of FACS-sorted microglia (CD45^low^/CD11b^+ve^/Ly6G^−ve^/Ly6C^−ve^) from WT and CCR2 KO SCI lesions four days (**d**) and seven days (**e**) after injury (*n =* 3–4, 4–5 mice pooled per *n*). (**d**) Graph shows the top 20 significantly down-regulated genes in WT animals compared with those genes in CCR2 KO mice. Each genotype was normalized to its own baseline control. (**e**) The same genes were assessed seven days after injury; several genes continue to show dysregulation compared with WT controls. Statistical analysis, Student *t* tests and one-way ANOVA. Mean ± SEM. *p < 0.05; **p < 0.01; ***p < 0.001. Corresponding raw data (**S1 Data**). CCR2, C–C chemokine receptor type 2; FAC, fluorescently activated cell sorting; KO, knock-out; MDM, monocyte-derived macrophage; RT-qPCR, quantitative real-time polymerase chain reaction; SCI, spinal cord injury; WT, wild-type.

To investigate this mechanism in vivo, we performed SCI in WT and *mpges −/−* mice and assessed the phagocytic microglial response. In addition, as our in vitro data show that PGE_2_ derived from macrophages suppresses microglial phagocytosis, we performed SCI in C–C chemokine receptor type 2 (CCR2) null mice to compare the microglial response in a lesion that contains very few MDMs (**see Fig 5A and 5B**) [33]. Three days after SCI in WT, *mpges −/−*, and CCR2 null mice, microglia were isolated from the lesion and ex vivo phagocytosis of pHrodo (Green)-myelin was quantified by flow cytometry **(Fig 4A and 4B)**. Microglia from WT mice after SCI (i.e., with macrophages and PGE_2_ present in the lesion), showed low levels of phagocytosis (**Fig 4A and 4B**). In contrast, microglia from *mpges −/−* and CCR2 null mice showed significantly increased phagocytosis (**Fig 4A and 4B**), indicating that in the absence of PGE_2_, or the absence of macrophages in the lesion, microglial phagocytic function is increased.

To assess the effect of blocking the EP2 receptor, in vivo, on microglial phagocytosis, we injected pHrodo-myelin into the brain (corpus callosum) of WT mice, together with vehicle or the EP2 receptor antagonist (PF-04418948). We assessed brain tissue three days after injection with immunofluorescence and confocal microscopy. In the corpus callosum of mice injected with pHrodo-myelin and vehicle, the area containing pHrodo-myelin is mainly populated with CD11b+, Tmem119-negative cells (MDMs) (**Fig 4C**). In mice injected with pHrodo-myelin and EP2 antagonist, there is a significant increase in Tmem119+ microglial cells in the area containing pHrodo-myelin (**Fig 4D and 4E**). Many of these Tmem119+ microglia contain or are closely associated with the fluorescently tagged myelin (**Fig 4D and 4F**). Importantly, the percentage of total microglial cells in the corpus callosum that are in contact with or contain pHrodo-myelin is significantly increased in EP2 antagonist–injected mice compared with controls (**Fig 4C, 4D and 4F**). These data indicate that blocking the EP2 receptor pathway in vivo promotes microglial phagocytic activity and increases recruitment of microglia to the site of injury. In summary, in vivo and in vitro studies suggest macrophage production of PGE_2_ acts at the EP2 receptor to mediate suppression of microglial phagocytosis.

### Macrophages influence microglial cell death but not proliferation or process extension towards lesion

We also sought to investigate macrophage effects on microglial cell death and proliferation, as microglia proliferate at the sites of CNS injury [14, 34]. In vitro, the presence of macrophages did not significantly reduce microglial viability (**S4A Fig**). However, when combined with inflammation (LPS stimulation), macrophages significantly reduced viability, suggesting an increase in microglial cell death, compared with untreated microglia (**S4A Fig**). This fits with our transcriptional profiling data, which show that apoptotic and cell death pathways are significantly dysregulated under similar conditions (**Fig 2B**). These data suggest that macrophages can affect microglial apoptosis under inflammatory conditions in vitro and warrant further analysis.

To investigate macrophage effects on microglial proliferation, we used Click-iT EdU assay (Invitrogen), which incorporates 5-ethynyl-2′-deoxyuridine (EdU; a nucleoside analog of thymidine) to DNA during active DNA synthesis. We found no evidence that macrophages affect the proliferation of microglia in vitro with bilaminar cultures (**S4B and S4C Fig**). We also depleted circulating macrophages prior to their infiltration after SCI using clodronate liposomes in LysM-eGFP mice to assess microglial proliferation with Ki67. Clodronate significantly depleted eGFP+ infiltrating cells at the lesion site when assessed five days after injury (**S4D and S4E Fig**), but this had no effect on microglial proliferation (**S4F and S4G Fig**).

To investigate whether macrophages modulated another important microglial function, namely rapid process extension towards microlesions, we induced laser lesions in organotypic hippocampal slice cultures (OHSCs), as done previously [35], in the presence or absence of BMDMs. We also investigated the initial reaction of microglial morphologies in an in vivo model of traumatic brain injury (TBI) [36] with local administration of Butaprost versus vehicle control. Initial process extension and acute morphological changes that are dependent on purinergic receptor signaling [6, 36] are not affected in OHSCs by the presence of macrophages (**S4H–S4M Fig**) or altered by the EP2 agonist in TBI (**S4N–S4O Fig)**.

In summary, coupled with our transcriptional profiling data, these results highlight that macrophages target specific pathways and functions in microglia, such as inflammation and apoptosis, but do not affect microglial proliferation or their rapid process extension response to injury. These findings may be of significance, as the rapid reaction of microglia in the early phases of injury are thought to be protective [35, 37, 38]. Our results suggest that peripheral macrophages do not interfere with this response.

### Blocking infiltration of macrophages into the CNS alters microglial inflammatory gene expression, increases chronic microglial activation, and impairs functional recovery after SCI

Mice that lack CCR2 cannot successfully recruit MDMs to traumatic CNS lesions [33]. We showed that this leads to increased microglial phagocytic activity (**Fig 4A and 4B**). Therefore, we next assessed whether lack of MDM infiltration affects activation of microglia and functional recovery in vivo after SCI using CCR2 null mice [33]. Although CCR2 has been reported to be expressed in injured neurons, expression appears variable between species and between investigators [39–41]. We are not aware of other genetic evidence for CCR2 protein expressed in neurons [13, 42]. CCR2 is also reported to be expressed in a subset of T-regulatory cells (Tregs) [43]. Although it is unknown what role CCR2+ Tregs play after SCI, it has been shown that T cells may play a beneficial role in CNS repair [44]. Despite this, a major phenotype five days after SCI was that MDMs were almost absent in the lesioned spinal cord of CCR2^rfp/rfp^ (CCR2 KO) mice, as compared with WT mice (**Fig 5A and 5B**). Neutrophil infiltration was not affected five days after injury (**Fig 5C**). Three days after injury, there was a trend to an increase, but this was not statistically significant (**S4P Fig**). To study the impact of the absence of MDMs on microglial-mediated inflammation, we isolated microglia from SCI lesions four and seven days after injury and assessed the expression profiles of 86 inflammatory genes using a PCR array. Four days after SCI in WT mice, approximately half of these genes were significantly down-regulated in microglia. Seven of the top 20 most down-regulated genes in WT mice were significantly less down-regulated in CCR2 KO mice, indicating that the absence of MDMs at the lesion resulted in less suppression of microglial inflammation (**Fig 5D**). Moreover, pathways identified as being suppressed by macrophages in our in vitro bilaminar system, such as inflammation driven by *MyD88* and *NF-κB* and apoptotic pathways driven by *Trp53* and *Bcl2* were also significantly more suppressed when MDMs were present at the lesion (**Fig 5D**). Seven days after SCI, microglial inflammatory genes continued to be dysregulated (**Fig 5E**). Components of important inflammatory pathways continued to be significantly less suppressed in the absence of macrophages, such as *MyD88*, *Il17a*, and *Cxcl2*. However, dysregulation of microglial gene expression was less unidirectional than factors associated with pro-inflammatory response, such as *Irf1*, *Tlr9*, and *Il12b*, and growth factors such as *Egf* and *Tgfb1* associated with recovery were significantly more down-regulated in microglia, in the absence of infiltrating macrophages (**Fig 5E**).

**Fig 6:**
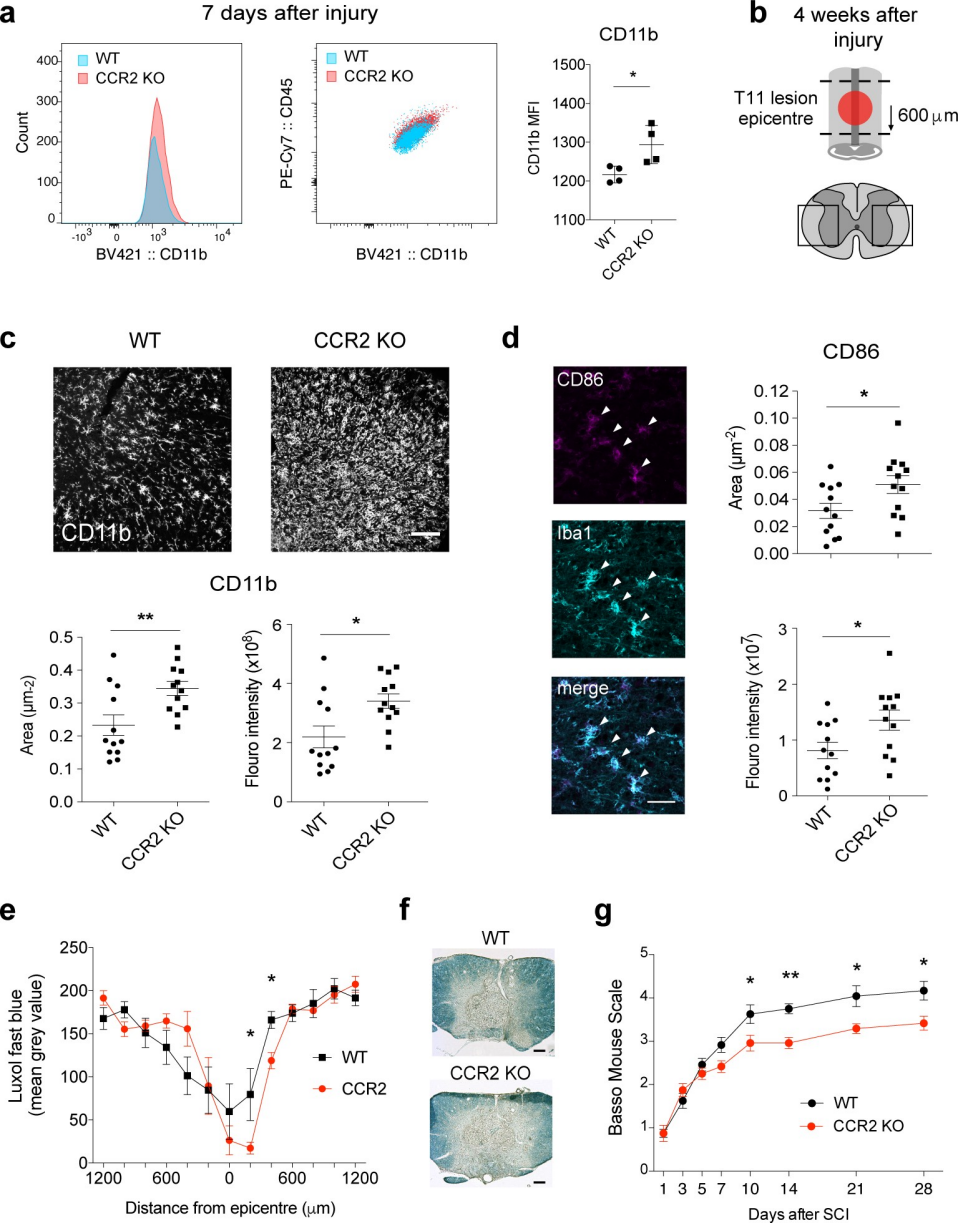
Blocking infiltration of macrophages to the injured spinal cord increases chronic microglial activation and impairs functional recovery after SCI. Spinal cord contusion injury was performed in CCR2 KO mice and WT controls. (**a**) FACS analysis of CD45^low^/CD11b^+ve^/Ly6G^−ve^/Ly6C^−ve^ microglia shows significant increase in CD11b mean fluorescent intensity (MFI) in CCR2 KO mice seven days after SCI. (**b**) Schematic of spinal cord shows area imaged and quantified in panels **c** and **d**, 28 days after injury—600 μm caudal to T11 lesion and boxed regions in left and right lateral white and gray matter. (**c**) Representative images in WT and CCR2 KO of CD11b immunoreactivity 28 days after SCI. Graphs show CD11b average area (μm^−2^) and fluorescent intensity (Integrated Density a.u) are significantly increased in CCR2 KO mice (which lack macrophage infiltration) versus WT controls. (**d**) CD86 expression (magenta) colocalized with Iba1 (cyan) is also significantly increased in CCR2 KO 28 days after SCI. (**e, f**) Luxol Fast Blue staining revealed that CCR2 KO mice showed greater myelin loss, a marker for secondary tissue damage, caudal to the lesion 28 days after SCI compared with control (**g**). Locomotor recovery assessed by the BMS shows CCR2 KO mice have impaired functional recovery compared to WT, beginning seven days after SCI. Statistical analysis for (**a, c, d, e**) Student *t* tests; **a**, *n* = 4; **c** and **d**, *n* = 12; **e**, *n* = 6 per group. (**g**) Two-way repeated measures ANOVA with post hoc Tukey analysis. *n =* 12 per group, mean ± SEM. *p < 0.05; **p < 0.01. Scale bars in **c** and **f** = 100 μm; **d** = 50 μm. Corresponding raw data (**S1 Data**). BMS, Basso Mouse Scale; CCR2, C–C chemokine receptor type 2; FACS, fluorescently activated cell sorting; KO, knock-out; MFI, mean fluorescent intensity; SCI, spinal cord injury; WT, wild-type.

Our previous work shows that initial perturbation to inflammatory networks is likely to cause unpredictable patterns of expression at later time points [24, 26]. Therefore, to investigate the long-term consequence of the initial loss of microglial suppression and subsequent dysregulation, we assessed long-term activation of microglia and its impact on functional recovery after SCI in CCR2 KO versus WT mice (**Fig 6A–6G**).

Up-regulation of CD11b (αM integrin) is well established as a readout of microglial/macrophage activation [45–47], and CD86 is a costimulatory receptor up-regulated during inflammation in microglia in vivo [48, 49]. CD11b expression in microglial cells was already increased at a cellular level in mice lacking macrophage infiltration (CCR2 KO) seven days after SCI versus controls (**Fig 6A**). There was a trend to an increase in CD86+ microglia but it did not reach statistical significance **(S4Q Fig).** To assess microglial activation 28 days after injury, we quantified CD11b and CD86 expression by immunofluorescence of tissue sections caudal to the lesion epicenter (**Fig 6C and 6D**). Importantly, 28 days after SCI, CD11b and CD86 immunoreactivity was greater in area and intensity in CCR2 KO mice despite the lack of infiltrating MDMs, which also express CD11b and CD86 (**Fig 6C and 6D**). In other words, CD11b and CD86 expression is markedly increased in microglia in CCR2 KO mice 28 days after SCI. These results indicate that preventing the communication between MDMs and resident microglia contribute to long-term microglial activation after CNS injury.

We also investigated whether increased microglial inflammation in the absence of infiltrating macrophages in CCR2 KO mice influences functional recovery and histopathology. CCR2 KO mice showed greater myelin loss, an indicator of secondary tissue damage, caudal to the lesion 28 days after SCI, compared with controls (**Fig 6E and 6F**). The increased microglial activation associated with the absence of macrophage influx after SCI is associated with worse locomotor recovery in CCR2 KO mice compared with WT controls, as measured by the Basso Mouse Scale (BMS) (**Fig 6g**).

## Discussion

The role of microglia in CNS injury and disease is now considered critical to the pathological process [50]. Our work suggests a novel concept that macrophages from the peripheral circulation, which enter the CNS after injury, may act to modulate microglial activation, thus preventing microglial-mediated acute and chronic inflammation.

These findings support previous work that shows blocking CCR2-dependent macrophage infiltration with an anti-CCR2 antibody worsens locomotor recovery after CNS injury [51, 52]. However, these earlier papers [51] did not show how macrophages mediate these effects. Our work now shows that infiltrating macrophages suppress microglial activation by reducing their expression of inflammatory molecules and ability to phagocytose, thus preventing chronic microglia-mediated inflammation in the CNS. Other work has suggested that subsets of infiltrating macrophages are detrimental to SCI [53], and it has been reported that CCR2 antagonism, producing a 50% reduction in infiltrating macrophages, is beneficial after TBI [54]. Also, CCR2 KO mice showed acute and transient behavioral improvement after intracerebral hemorrhage (one and three days), but this was not sustained at seven days [55]. Here, our finding that inhibition of macrophage entry to the CNS results in a worse outcome after SCI is supported by work that defines specific beneficial macrophage populations in multiple CNS injury and disease contexts [56–59]. Our results now suggest a new mechanism by which infiltrating macrophages mediate their beneficial actions via the regulation of microglial activation. Such a mechanism will operate alongside macrophage-intrinsic mechanisms. We observed that macrophages regulate microglia in both mouse and human cells. This is important as it represents an independent replication of the concept in a different laboratory. It shows that macrophages derived from the blood (human) or bone marrow (mouse) appear to have similar effects on microglia and that the findings may be relevant to human disease.

It is still controversial as to whether the net effect of microglia is beneficial or detrimental to CNS injury [4, 5, 60]; however, the kinetics of the microglial response must be considered. There is evidence that the initial responses of microglia, which occur in the first few minutes to several hours after injury, are beneficial and limit the expansion of CNS lesions [35, 37, 38]. Conversely, prolonged microglial dysregulation and neuroinflammation are deleterious to the CNS [61]. Therefore, the initial microglial response to injury may be beneficial, but prolonged inflammation and activation are potentially detrimental to recovery. Our data suggest that macrophages play a role in mitigating this detrimental response by infiltrating the injury site and reducing microglial-mediated inflammation and chronic microglial activation. The absence of this protective mechanism may contribute to a worse outcome when infiltrating macrophages do not enter the CNS after SCI in CCR2 KO mice. To our knowledge, this is the first description of such a cellular mechanism to reduce deleterious consequences of CNS injury.

Microglial cells are now prime targets in drug discovery for CNS injuries and neurodegenerative diseases [62]. To properly assess the roles of microglia in CNS injury, our data suggest that the context, timing, and interaction with macrophages should also be considered. Attempts to target either of these two cell populations should be approached with caution and a better understanding is needed of their divergent and complex roles in injury and disease. The heterogeneity and region-specific differences in microglia [63] and macrophage populations [64] will also need to be considered.

Recent work has shown that peripherally derived macrophages can engraft the brain and maintain an identity distinct from microglia [65], thus opening the possibility for therapeutic engraftment of MDMs to the CNS and allowing macrophage–microglia cross talk in disease contexts. Cell-to-cell interactions between different brain resident cell types are now becoming evident [66–69]. During inflammation, microglia have also been shown to drive astrocyte-mediated toxicity [66], which, subject to the context, is dependent on microglial NF-κB signaling [67]. Our data show that peripheral macrophages regulate the NF-κB signaling pathway in microglia that, in turn, reduce inflammatory mediators, such as TNF, which can drive astrocyte-mediated toxicity [66, 67]. This raises the possibility that macrophage signaling to microglia may have subsequent effects in other CNS cells, such as astrocytes.

In summary, we suggest that infiltrating macrophages provide a natural control mechanism against detrimental acute and long-term microglial-mediated inflammation. Manipulation of peripherally derived infiltrating cells may provide a therapeutic treatment option to target microglial-mediated mechanisms that cause or exacerbate CNS injury and disease.

## Materials and methods

### Ethics statement

All animal procedures were approved by the Animal Care Committee of the Research Institute of the McGill University Health Centre and followed the guidelines of the Canadian Council on Animal Care and the ARRIVE guidelines for reporting animal research [70]. Before surgical interventions and cardiac perfusions, mice were deeply anesthetized by intraperitoneal injection of ketamine (50 mg/kg), xylazine (5 mg/kg), and acepromazine (1 mg/kg). Human brain tissue was collected during clinical practice, fully anonymized, and therefore available for use under the legislation of the Tri-Council Policy Statement two and Plan d'action ministériel en éthique de la reserche et en intégrité scientifique of Quebec and Canada. This study was carried out in accordance with the guidelines set by the Biomedical Ethics Unit of McGill University, approved under reference ANTJ2001/1, and conducted in accordance with the Helsinki Declaration.

### Strains of mice used

C57BL/6 (Charles River, St-Constant, QC), heterozygote lysM^+/EGFP^ mice (kindly provided by Dr. Thomas Graf and obtained from Dr. Steve Lacroix); homozygote CCR2^RFP/RFP^ and their C57BL/6J controls (Jackson); heterozygote Cx3CR1^+/gfp^ (Jackson) and Ptges^−/−^ mice [71] (obtained from Dr. Maziar Divangahi, McGill University), aged 8–14 weeks, were kept under a 12-hour light/dark cycle with ad libitum access to food and water. The LysM-eGFP mouse was originally generated by Faust and colleagues, 2000. EGFP is expressed specifically in the myelomonocytic lineage by using homologous recombination. This was achieved by knocking the enhanced GFP (EGFP) gene into the murine lysozyme M (lys) locus and using a targeting vector, which contains a neomycin resistant (neo) gene flanked by LoxP sites and “splinked” ends, to increase the frequency of homologous recombination. Removal of the neo gene through breeding of the mice with the Cre-deleter strain led to an increased fluorescence intensity [22].

### Spinal cord contusion

Female mice were anesthetized by intraperitoneal injection of ketamine (50 mg/kg), xylazine (5 mg/kg), and acepromazine (1 mg/kg) and a moderate contusion injury (50 kDa force; 500–600-μm tissue displacement) was made at the T11 thoracic vertebral level using the Infinite Horizon Impactor device (Precision Scientific Instrumentation, Lexington, KY), as previously described [72].

### Isolation of adult mouse microglia for cell culture

Male C57BL/6 mice (8–12 weeks) were, anesthetized, transcardially perfused and brains removed and kept in ice-cold Hanks Balanced Salt Solution (HBSS). Cerebellum and meninges were removed, and brain was cut into small pieces. Tissue was enzymatically dissociated using Neural Tissue Dissociation Kit (P) (Miltenyi cat # 130-092-628) according to the manufacturer’s instructions, with modifications. Following digestion, tissue was transferred to a 15-mL Dounce on ice and homogenized with 20× passes of a large clearance pestle. Tissue was resuspended in 35% isotonic percoll and overlaid with HBSS. Following centrifugation (400*g*; 45 minutes), myelin was removed, and pure populations of microglial cells were selected using CD11b microbeads (Miltenyi #130-093-634), as previously described [63, 73]. Pure (>95% CD11b-positive) adult microglia were resuspended at 8×10^5^ cell^−mL^ (approximately two brains per mL) in media (DMEM F12, 10% fetal bovine serum [FBS], 1% penicillin/streptomycin [P/S]), with 10% L-cell conditioned media, a source of macrophage colony-stimulating factor (M-CSF), or 10 ng/mL recombinant mouse M-CSF (R and D cat no 416-ML-010/CF), and 50 ng/mL recombinant human TGF-β1 (Miltenyi cat no: 130-095-067) to maintain their transcriptional profile, as previously described [11]. Cells were plated in pre-coated poly-L-lysine plates, media was changed at three days, and experiments were performed at seven days. At seven days, microglia were collected and microglial “signature” genes assessed by qPCR. Network analysis and Markov clustering (see below) were performed to assess conditions driving cells to a similar phenotype of their freshly isolated counterparts.

### Ex vivo phagocytosis assay

CD11b^+^ cells (myeloid cells) were collected by magnetic bead cell sorting, as above, from SCI lesions of lys-EGFP-ki mice (2.5 mm either side of the epicenter) at 1 or 3 days after injury, or from uninjured controls. The CD11b^+^ fraction was immediately plated into 96 well plates (pre-coated poly-L-lysine, one animal per well) in DMEM F12 media containing 10% FBS. Cells were incubated for four hours with pHrodo (Invitrogen)-labeled myelin and taken for FACS analysis.

### BMDMs

BMDMs were generated as previously described [74] from adult male C57/BL6− or Ptges^−/−^ mice. Mice were euthanized, and their femurs were removed. Bone marrow was flushed out and homogenized, and RBCs were hypotonically lysed. After washing, cells were cultured in RPMI media containing 10% FBS, 10% L-cell-conditioned media, and 1% P/S for seven days.

### Bilaminar cultures

Adult microglia and BMDMs (from the same animal when possible) were cultured separately for seven days, as described above; BMDMs were then replated on poly-L-lysine–coated glass coverslips. Cells were plated at 2×10^5^ per 25-mm coverslip (for insertion in 6 well plates) of 4×10^4^ per 12-mm coverslip (for insertion in 24 well plates). These numbers were calculated to match the number of microglia in the same well as, three days after SCI in vivo, the proportion of microglia to macrophages around the lesion (54% ± 8% versus 46% ± 8%). Therefore, equal numbers of microglia and BMDMs were plated into wells in vitro. Coverslips were pre-mounted with 3 small paraffin droplets on the same surface as the cells were plated, as previously described for neuron-astrocyte cocultures [75]. BMDMs were allowed to adhere overnight. Bilaminar culture experiments began by inserting coverslips containing BMDM and wax paraffin nodule face down into culture plates containing adult microglia. All experiments were performed in DMEMF12 serum-free media.

### In vitro phagocytosis assays

Adult microglia and BMDMs were cocultured in the bilaminar system, as above, for 24 hours. At 24 hours, BMDM coverslips were removed from the microglial wells and placed in new cell culture wells. Following separation, both adult microglia and BMDMs were incubated with pHrodo Red (ThermoFisher, Mississauga, ON) labeled myelin for 90 minutes. pHrodo Red is weakly fluorescent at neutral pH but increasingly fluorescent as the pH drops, therefore an increasing signal can be detected because of pHrodo-myelin entering the lysosome. After treatment, cells were trypsinized, harvested, and labeled for FACS analysis.

### In vitro inflammatory stimulation

Adult microglia and BMDMs were cocultured in the bilaminar system, as above. Wells containing microglia and/or BMDMs were treated with vehicle or LPS (100 ng/mL) for four hours. Cells were then washed and fresh, serum-free media was added to each well for a further 20 hours. At 24 hours, supernatants were harvested and cells lysed with 350 μL of RLT lysis buffer (Qaigen, Germantown, MD) and snap frozen until RNA extraction. For the in vitro proliferation assay, microglia rather than BMDMs were plated on coverslips. Cells were treated as above, with the addition of Click-iT EdU (20 μM) at the time of LPS administration. EdU is a nucleoside analog of thymidine that is incorporated into DNA during active DNA synthesis and was used according to the manufacturer’s instructions (Invitrogen, Carlsbad, CA).

### Human myeloid cells

To obtain MDMs, monocytes were isolated from healthy human venous blood. PBMCs were isolated from whole blood using Ficoll–Paque density gradient centrifugation (GE Healthcare, Piscataway, NJ). CD14+ monocyte isolation was done using immune-magnetic bead selection according to the manufacturer’s instructions to achieve 95%–99% purity (Miltenyi Biotec), as determined by flow cytometry. Cells were cultured in RPMI (Invitrogen, Carlsbad, CA) supplemented with 10% FBS, 0.1% P/S, and 0.1% L-glutamine. Cells were plated at a density of 5×10^5^ cells/mL in 6-well tissue culture plates for four days and matured in vitro to become MDMs, using supplementation with recombinant M-CSF (25 ng/mL, PeproTech, Rocky Hill, NJ).

Human microglia were isolated from adult brain tissue using previously described protocols [76]. Adult microglia were derived from surgical resections of brain tissue from pharmacologically intractable nonmalignant cases of temporal lobe epilepsy. The tissue provided was outside of the suspected focal site of epilepsy-related pathology. Briefly, tissue was obtained in pieces <1 mm^3^ and treated with DNase (Roche, Nutley, NJ) and trypsin (Invitrogen, Carlsbad, CA) for 30 min at 37 °C. Following dissociation through a nylon mesh (37 μm), the cell suspension was separated on a 30% Percoll gradient (GE Healthcare, Piscataway, NJ) at 31,000*g* for 30 minutes. Glial cells (oligodendrocytes and microglia) were collected from underneath the myelin layer, washed, and plated at a density of 2×10^6^ cells/mL in tissue culture flasks. After 24 hours in culture, microglia were separated by the differential adhesion properties of the cells. Microglia were grown for four days in flasks before gently collecting using 2 mM EDTA (Sigma-Aldrich, Oakville, ON); cells were then plated in minimum essential medium (MEM, Sigma-Aldrich, Oakville, ON) supplemented with 5% FBS, 0.1% P/S, and 0.1% L-glutamine at a density of 5×10^5^ cells/mL on a glass 25-mm cell culture insert in a 6-well plate.

Following addition of microglia to the MDM-containing well, as described above in the bilaminar coculture system, cells were exposed to 100 ng/mL lipopolysaccharide (serotype 0127:B8, Sigma-Aldrich) for 24 hours before collection in Trizol Reagent (Invitrogen, Carlsbad, CA) for subsequent total RNA isolation using the Qiagen RNeasy mini kit following the manufacturer's instructions. RNA isolated was treated immediately with DNase (Qiagen, Germantown, MD). Reverse transcription and cDNA generation were performed using random hexaprimers (Roche) and the Moloney murine leukemia virus-RT enzyme (Invitrogen, Carlsbad, CA) at 42 °C. PCR reaction cycling was performed according to the ABI PRISM 7000 Sequence Detection System default temperature settings (two minutes at 50 °C, 10 minutes at 95 °C, followed by 40 cycles of 15 seconds at 95 °C, one minute at 60 °C).

TaqMan quantitative real-time PCR was used to measure mRNA expression levels for all mRNAs. Relative gene expression data were calculated according to the 2^−*ΔΔCt*^ method.

### Drug treatments

For phagocytosis assay after treatment with EP2 receptor agonist, adult microglia were treated for one hour with Butaprost (1 μM) (Cayman Chemicals, Ann Arbor, MI) prior to incubation with pHrodo-myelin. For treatment with EP2 antagonist, bilaminar cocultures of adult microglia and BMDMs were treated with PF-04418948, (10 μM) (Sigma-Aldrich, Oakville, ON) prior to incubation with pHrodo-myelin. For treatment of adult microglia or BMDMs with Butaprost during inflammation, wells containing microglia or BMDMs were treated with vehicle or LPS (100 ng/mL) and Butaprost (1 μM) for four hours. Cells were then washed and fresh, serum-free media containing Butaprost (1 μM) was added to each well for a further 20 hours. At 24 hours, supernatants were harvested and cells lysed with 350 μL of RLT lysis buffer (Qaigen, Germantown, MD) and snap frozen until RNA extraction.

### Cortical injection of myelin and EP2 antagonist

Injections were made into the right motor cortex of eight-week-old female C57/BL6J mice. A 2 × 2 mm opening was made in the skull just to the right side of the midline and just below bregma. A 26G needle attached to a 10-μL World Precision Instruments NanoFil syringe was used. The needle was inserted into the cortex just deep enough for the entire bevel of the needle to be inside the brain—a depth of approximately 0.46 mm. The EP2 receptor antagonist (PF-04418948) (Sigma-Aldrich, Oakville, ON) was initially dissolved in 100% DMSO to a 50-mM stock solution. This solution was diluted in PBS, pH 8.0, for a final injection concentration of 1 uM. The vehicle controls were a corresponding dilution of DMSO in PBS, pH 8.0. The total volume injected was 2 μL. Each injection contained 20 μg of pHrodo-labeled myelin. The pHrodo-myelin was prepared as follows: 18.5 μL myelin (15 mg/mL) + 25 μL of pHrodo was suspended in 206.5 μL of PBS, pH 8.0, and incubated 45 minutes at RT in the dark on the rocker. Samples were then spun down at 4000*g* for 10 minutes and resuspended in 25 μL of PBS plus either vehicle or antagonist. Three days after injection, mice were humanely killed and transcardially perfused, as described below.

### OHSC and laser lesions

Cultures were prepared as previously described [77]. Briefly, 300-μm hippocampal slices were prepared from P6-7 CX_3_CR1^gfp/+^ mice and cultured on semiporous nylon membranes (Millipore, Bedford, MA) for 9–11 days, with medium changes every two days. The culture medium contained 6.5 mg/mL glucose, 25% HBSS, 25% horse serum, 50% minimum essential medium with Glutamax, and 0.5% P/S (Gibco, Mississauga, ON). Five experimental conditions were assessed: (1) control slices in serum-free slice culture medium, (2) OHSCs incubated with a feeder layer of BMDMs for 24–36 hours, (3) OHSCs incubated with a feeder layer of BMDMs pre-stimulated with LPS (100 ng/mL, two hours) for 24–36 hours, (4) OHSCs incubated with BMDMs added directly onto the slice (5×10^5^/slice), for 24–36 hours, and (5) OHSCs incubated with a LPS pre-stimulated (100 ng/mL, two hours) BMDMs added directly onto the slice (5×10^5^/slice) for 24–36 hours prior to laser lesioning.

Laser lesions and imaging of microglial responses were performed with a customized Olympus FV1200MPE equipped with a MaiTai HP DeepSee-OL IR laser and a 25× XLPlan N objective. Slice cultures were perfused at 1–2 mL/minute with room temperature artificial cerebral spinal fluid containing (in mM) NaCl (126), NaHCO_3_ (26), KCl (2.5), NaH_2_PO_4_ (1.25), glucose (10), MgCl_2_ (2), and CaCl_2_ (2), corrected for osmolarity with sucrose. Laser lesions and imaging were performed sequentially at 820 nm, which provided excitation of both GFP and lipofuscin [35]. Lesions were performed in a 3-μm circular ROI at a depth of 40–70 μm in the central plane of the imaged volume, using 2–7 scans at 60% laser power, with a pixel dwell time of 10 μs. Post-lesion images were 30-μm z-stacks with a 1.5-μm step size, once per minute for 15 minutes. Analysis was performed with MTrackJ for ImageJ [78].

### Compression injury and intravital two-photon microscopy

For mTBI experiments, CX_3_CR1^gfp/+^ mice were anesthetized with ketamine, xylazine, and acepromazine and maintained at a core temperature of 37 ºC. Hair was removed from the head using hair clippers and Nair. An incision was made in the scalp to expose the skull and a metal bracket was secured on the skull bone over the barrel cortex. The bone was quickly thinned to a thickness of about 20–30 μm. Once thinned, the blunt end of a microsurgical blade was used to compress the skull bone into a concavity without cracking the skull. Artificial spinal fluid (ACSF) or 1 μM Butaprost (Cayman Chemicals, Ann Arbor, MI) in ACSF was immediately applied to the skull after mTBI and kept on throughout the imaging experiment. Mice that had mTBI procedures were imaged using a Leica SP8 two-photon microscope equipped with a 12,000-Hz resonant scanner, a 25× color corrected water-dipping objective (1.0 NA), a quad HyD external detector array, and a Mai Tai HP DeepSee Laser (Spectra-Physics, Santa Clara, CA) tuned to 905 nm. Three-dimensional time-lapse movies were captured in z-stacks of 15–30 planes (3-μm step size) at 1–2-minute intervals. Signal contrast was enhanced by averaging 10–12 video frames per plane in resonance scanning mode. Three-dimensional time-lapse movies were imported for analysis into Imaris software (Bitplane). “Jellyfish” microglia were quantified by counting cells that had a process that was 20 μm or more in diameter at any time within about two hours of imaging immediately following mTBI. This number was then divided by the area of reactive microglia for each mouse.

### Clodronate liposomes

Clodronate liposomes used to deplete the population of infiltrating macrophages were purchased through the website, www.clodronateliposomes.com, and were prepared by Nico Van Rooijen in the Netherlands. Clodronate liposomes were injected on the day of spinal cord contusion and at days 1, 2, 3, and 4 after the injury. For each treatment, 100 μL/10 g body weight was administered intravenously (IV) and 50 μL/10 g intraperitoneally (IP). Saline injections were administered to control animals at the equivalent time.

### Flow cytometry

For analysis of cells from ex vivo experiments, cells were harvested with trypsin and stained with eflouro eFluor780 viability dye (1:1,000; eBioscience, Mississauga, ON), blocked with FC-receptor blocked (1:200; BD Bioscience), and stained with CD11b-V450, Ly6G-PerCP-Cy5.5 (all 1:200; BD Bioscience, Mississauga, ON). LysM-GFP and pHrodo Red labeled myelin were detected in the FITC and PE channels, respectively. CD11b^+^/Ly6G^−^/LysM-GFP^−^ (microglia) CD11b^+^/Ly6G^−^/LysM-GFP^+^ (macrophages) were assessed for pHrodo-labeled myelin; the content was detected in the PE channel. For analysis of cells from in vitro experiments, adult microglia or BMDMs were harvested with trypsin and stained with eflouro eFluor780 viability dye (1:1,000; eBioscience, Mississauga, ON), blocked with FC-receptor blocked (1:200; BD Bioscience), and stained with CD11b-V450 (1:200; BD Bioscience, Mississauga, ON). pHrodo-labeled myelin was detected in the PE channel.

For analysis of cells from in vivo experiments, spinal cord tissue was harvested and transferred to a 15-mL Dounce homogenizer on ice and homogenized with 20× passes of a large clearance pestle. Tissue was resuspended in 70% and overlaid 30% isotonic Percoll. Following centrifugation (900*g*; 25 minutes), cells at the Percoll interface were collected, washed, and resuspended. Cells were blocked with FC-receptor blocked (1:200; BD Bioscience, Mississauga, ON) and stained with CD45-PE-Cy7, CD11b-V450, Ly6G-APC, or FITC, Ly6C-APC-Cy7 (all 1:200; BD Bioscience, Mississauga, ON). CCR2-RFP was detected in the PE channel. Cells were acquired using a BD FACS Canto II and analyzed using FlowJo software. For fluorescently activated cell sorting (FACS), a BD FACSAria Fusion (BD Bioscience, Mississauga, ON) was used to isolate CD11b^+ve^/CD45^lo^/Ly6G^−ve^/Ly6C^−ve^ microglial cells, which were directly sorted in Trizol for subsequent RNA extraction. All gating strategies not contained within the figures are presented in the Supporting information (**S5 Fig**).

### RNA extraction from FACS-sorted microglia

Total RNA from FACS-sorted microglia was extracted from WT and CCR2-KO spinal cord injured mice, four and seven days after injury, or genotype-matched uninjured controls, as described previously [79]. Briefly, CD45^hi^/CD11b^+ve^/Ly6G^−ve^/Ly6C^−ve^ microglial cells were recovered in 700 μL of Trizol, vortexed, centrifuged, snap frozen in dry ice, and kept at −80 °C until further use. On the day of extraction, samples were thawed and 140 μL of chloroform (Fisher) was added, vortexed for 15 seconds, and centrifuged at 12,000*g* at 4 °C for 15 minutes. The clear aqueous layer was recovered and 1 μL of Glycoblue (20 mg mL–1, Ambion; AM9515) was added, followed by 1:1 volume of isopropanol (Thermofisher Mississauga, ON) (approximately 400 uL). Samples were kept overnight at −20 °C to precipitate the RNA. The next day, the samples were centrifuged at maximum speed for 20 minutes at 4 °C. At this point, a blue pellet of RNA is visible. The pellet was washed twice with 75% RNAse-free ethanol; it was let dry at room temperature and resuspended in 14 μL of RNAse-free H_2_O. To assess inflammatory and immune-related genes, an RT^2^ Profiler PCR array was used (Qiagen; PAMM-181Z), following instructions provided by the manufacturer. This array screened for 84 genes, and the data obtained were analyzed with the online Qiagen analysis software (RT2 profiler PCR array data analysis V3.5), http://www.qiagen.com/shop/genes-and-pathways/data-analysis-center-overview-page/.

### Quantitative real-time polymerase chain reaction

We performed quantitative real-time polymerase chain reactions (RT-qPCRs) to assay for the expression levels of multiple transcripts. RNA was extracted from cultured adult microglial BMDMs using the RNeasy Mini Kit (Qiagen, Germantown, MD). For spinal cord tissue, samples were homogenized, and total RNA was extracted using the RNeasy Lipid Tissue Kit (Qiagen, Germantown, MD). Reverse transcription was performed with the Quantinova Reverse Transcription Kit (Qiagen, Germantown, MD), and qPCR was performed using 1 μL of cDNA with Fast SYBR Green Master Mix (Applied Biosystems, CA) on a Step-One Plus qPCR machine (Applied Biosystems). Peptidylprolyl isomerase A (PPIA) was used as an internal control gene. The 2^−*ΔΔCt*^ method was used to calculate the cDNA expression fold change following standardization relative to PPIA [24, 80]. All primers had a Tm of 60°C. Primer sequences were as follows:

Mouse

p2ry12 forward: 5′ CTG GGA CAA ACA AGA AGA AAG G 3′

p2ry12 reverse: 5′ CCT TGG AGC AGT CTG GAT ATT 3′

mertk forward: 5′ CCT CCA CAC CTT CCT GTT ATA TT 3′

mertk reverse: 5′ TGT TGC TCA GAT ACT CCA TTC C 3′

itgb5 forward: 5′ GGA TCA GCC AGA AGA CCT TAA T 3′

itgb5 reverse: 5′ AAT CTT CAG ACC CTC ACA CTT C 3′

gas6 forward: 5′ AGG AGA CAG TCA AGG CAA AC 3′

gas6 reverse: 5′ TTG AGC CTG TAG GTA GCA AAT C 3′

fcrls forward: 5′ AAT CAC ATT CTC CTG GCA TAG G 3′

fcrls reverse: 5′ GCA TGG CTT TCC CTG ATA GT 3′

c1q forward: 5′ GAA AGG CAA TCC AGG CAA TAT C 3′

c1q reverse: 5′ GGT GAG GAC CTT GTC AAA GAT 3′

Tnf forward: 5′ TTG CTC TGT GAA GGG AAT GG 3′

Tnf reverse: 5′ GGC TCT GAG GAG TAG ACA ATA AAG 3′

Il6 forward: 5′ CTT CCA TCC AGT TGC CTT CT 3′

Il6 reverse: 5′ CTC CGA CTT GTG AAG TGG TAT AG 3′

Il1b forward: 5′ ATG GGC AAC CAC TTA CCT ATT T 3′

Il1b reverse: 5′ GTT CTA GAG AGT GCT GCC TAA TG 3′

Il10 forward: 5′ ACA GCC GGG AAG ACA ATA AC 3′

Il10 reverse: 5′ CAG CTG GTC CTT TGT TTG AAA G 3′

Ptges forward: 5′ CCA CAC TCC CTC TTA ACC ATA AA 3′

Ptges reverse: 5′ GCC AGA ATT GTA GGT AGG TCT G 3′

EP1 forward: 5′ CTC TCG ACG ATT CCG AAA GAC 3′

EP1 reverse: 5′ GTG GCT GAA GTG ATG GAT GA 3′

EP2 forward: 5′ GCC TTT CAC AAT CTT TGC CTA CAT-3′

EP2 reverse: 5′ GAC CGG TGG CCT AAG TAT GG-3′

EP4 forward: 5′ CAA GCA TGT CCT GTT GCT TAA C 3′

EP4 reverse: 5′ GTC GGT TCA GCT ACG CTT TA 3′

### Whole-transcript expression analysis

Total RNA was quantified using a NanoDrop Spectrophotometer ND-1000 (NanoDrop Technologies) and its integrity was assessed using a 2100 Bioanalyzer (Agilent Technologies). Sense-strand cDNA was synthesized from 100 ng of total RNA, and fragmentation and labeling were performed to produce ssDNA with the Affymetrix GeneChip WT Terminal Labeling Kit according to the manufacturer’s instructions (ThermoFisher-Affymetrix, Mississauga, ON). After fragmentation and labeling, 3.5 μg DNA target was hybridized on Mouse Clariom S Assay (ThermoFisher-Affymetrix) and incubated at 450 °C in the Genechip Hybridization oven 640 (ThermoFisher-Affymetrix) for 17 hours at 60 rpm. Clariom S were then washed in a GeneChips Fluidics Station 450 (ThermoFisher-Affymetrix) using Affymetrix Hybridization Wash and Stain kit according to the manufacturer’s instructions (ThermoFisher-Affymetrix). The microarrays were finally scanned on a GeneChip scanner 3000 (Affymetrix).

### Computational analysis and bioinformatics

Microarray data sets were normalized by the robust multiarray averaging (RMA) method in Affymetrix Expression Console (Affymetrix). To assess whether there were transcripts differentially expressed between activated microglia and activated microglia in the presence of macrophages, normalized data sets were compared in Affymetrix Transcriptome Analysis Console (TAC) Software. To assess whether there were transcripts differentially expressed between LPS-stimulated microglia and LPS-stimulated microglia in the presence of macrophages, normalized data sets were compared by ANOVA with a *p* < 0.05 cutoff. Differentially regulated gene lists were then assessed with the IPA software tool (Qiagen). To investigate gene co-expression relationships between groups, a pairwise transcript-to-transcript matrix was calculated in Miru from the set of differentially expressed transcripts using a Pearson correlation threshold *r* = 0.86. A network graph was generated in which nodes represent individual probe sets (transcripts/genes), and edges between them the correlation of expression pattern, with Pearson correlation coefficients above the selected threshold. The graph was clustered into discrete groups of transcripts sharing similar expression profiles using the MCL algorithm (inflation, 2.2; minimum cluster size, 10 nodes).

### Locomotor assessment

After SPI in mice, locomotor recovery was evaluated in an open field test using the 9-point BMS [81]. The BMS analysis of hind limb movements and coordination was performed by two individuals who were trained in the Basso laboratory, and the consensus score was taken. The final score is presented as mean ± SEM. The 11-point BMS subscore was also assessed.

### Immunofluorescence and histology

Animals were perfused with 4% paraformaldehyde in 0.1 M PBS, pH 7.4, at three, five and 28 days. Spinal cord segments containing the lesion site, or whole brain from pHrodo-myelin injected mice, were removed and processed for cryostat sectioning (20-μm-thick cross sections). Immunofluorescence was performed using rat anti-CD11b (1:250; Serotec, Raleigh, NC), rabbit anti Iba1 (1:500; Wako, Richmond, VA), anti-rat CD86 (1:200; BD Bioscience, Mississauga, ON), rabbit anti-P2ry12 (1:500; kindly provided by Dr. Oleg Butovsky), rabbit anti-Tmem119 (1:1 hybridoma supernatant; kindly provided by Dr. Mariko Bennett, Ben Barres Lab), rabbit anti-Ki67 (1:500; Abcam, Cambridge, MA), and chicken anti-GFP (1:500; Abcam, Cambridge, MA) and detected using the appropriate secondary antibodies at 1:500; anti-rabbit Alexa Fluor 568 or 647, anti-rat Alexa Fluor 568 or 647, and anti-chicken Alexa Fluor 488 (Invitrogen, Carlsbad, CA). All images were visualized using a confocal laser scanning microscope (FluoView FV1000; Olympus) using FV10-ASW 3.0 software (Olympus) and prepared with ImageJ. Histochemical staining with Luxol fast blue was used to assess myelin loss 28 days after injury. Myelin was quantified as a measure of Luxol fast blue (mean gray value, ImageJ) across the whole cross section, measured at 200-μm intervals over the 2-mm length of the cord.

### Quantification of immunofluorescence data

In spinal cord sections 28 days after injury, single plane images at a depth of 8 μm were acquired at two sites either side of the midline and included white and gray matter, as depicted in Fig 4. Images were taken 600 μm caudal to the lesion epicenter to avoid overt tissue cavities but include putative areas of MDM and microglia cell interactions. Images were acquired and analyses performed whilst blinded to genotype. Area of fluorescence and fluorescence intensity (as measured by Integrated Density [IntDen], which is the product of area and mean gray value) were quantified with ImageJ. For quantification of pHrodo-myelin injected mice, images were acquired from three consecutive slices around the injection site and analyses performed whilst blinded to treatment. The corpus callosum provided a boundary at which microglia cells and their association with pHrodo were quantified.

### Raw data files

All relevant data are within the paper and its Supporting information files (**S1 Data**). Microarray data are available at Gene Expression Omnibus (accession number GSE102482).

### Statistics

Data were analyzed using one-way and two-way ANOVAs with Bonferroni correction or Student *t* tests when appropriate and as indicated. Data were checked for compliance with statistical assumptions for each test, including normal distribution and equal variances across groups. Tests were two-tailed throughout. Statistical significance was considered at *p* < 0.05. Data show mean ± SEM.

## Supporting information

S1 Fig Microglia and infiltrating macrophages can be distinguished from each other by the expression of different proteins at the site of CNS injury; and infiltration of macrophages increases over time after SCI. (**a**) Confocal z-stack images of infiltrating macrophages (green) (CD11b^+ve^/LysM-eGFP^+ve^/Tmem119^−ve^; top panel) and (CD11b^+ve^/LysM-eGFP^+ve^/P2ry12^−ve^; bottom panel) in position to interact with Tmem119^+ve^ or P2ry12^+ve^ microglial cells (red). Scale bar = 20 μm. (**b**) Increasing numbers of MDMs in the injured spinal cord after SCI. Myeloid cells were isolated from the uninjured or injured spinal cord of LysM-eGFP mice and LysM-eGFP^+ve^ (macrophages) were quantified as a percentage of total CD45^+^/Ly6G^−ve^/CD11b^+^ cells. Statistics: One-way ANOVA with Bonferroni corrections (*n =* 3). Mean ± SEM. **p < 0.01; ***p < 0.001. Corresponding raw data (**S1 Data**). CNS, central nervous system; MDM, monocyte-derived macrophage; SCI, spinal cord injury.
(TIF)

S2 Fig Characterization of optimum culture conditions for adult mouse microglia and the development of an in vitro system to assess macrophage–microglial interactions. Primary adult mouse microglia were cultured under specific conditions in order to retain a transcriptional profile similar to in vivo adult microglia [11]. (**a**) Gene expression of seven microglia “signature” genes [11] in microglial cells cultured for seven days in DMEM-F12 media containing FBS (10%) (NM), with combinations of recombinant M-CSF (10 ng/mL), conditioned media from L-929 cells (rich in M-CSF; 10%) and recombinant human TGF-β1 (50 ng/mL). Fold changes are expressed relative to freshly isolated adult microglia. (**b**) Network graph showing sample-to-sample correlation of microglial signature gene expression shown in (**a**); analysis performed in Miru (Pearson correlation threshold, *r* ≥ 0.85). Nodes represent individual samples and edges the degree of correlation between them. The network graph was clustered using a Markov clustering algorithm, and samples were assigned a color according to cluster membership. Gene expression in freshly isolated microglia (brown) is most closely correlated with cultured microglia that had been treated with a source of M-CSF and TGF-β1 (purple). (**c**) Representative images of adult microglial cultures at seven days in the presence of L-929 conditioned media without and with TGF-β. (**d**) Schematic showing the bilaminar cultures. BMDMs were plated on coverslips on which small paraffin pegs were placed. These coverslips were then placed into wells containing adult microglia such that the two cell types were separated. (**e**) Adult mouse microglial gene expression of four microglia “signature” genes treated with LPS (100 ng/mL) in the presence or absence of macrophages (Mϕ). (**f**) Adult mouse microglia cultured with or without adult microglia and stimulated with LPS (100 ng/mL) show no difference in mRNA expression of IL-1β, TNF, and IL-6. Expression in microglia cultured alone is also shown. Statistical analysis; two-way ANOVA with Bonferroni corrections (*n =* 3–6), mean ± SEM. *p < 0.05; **p < 0.01; ***p < 0.001. Corresponding raw data (**S1 Data**). BMDM, bone marrow–derived macrophage.
(TIF)

S3 Fig Prostaglandin pathway regulation in mouse adult microglia and EP1, 2, and 4 receptor expression in mouse and human bilaminar cocultures and EP2 receptor agonists effects on BMDMs. (**a**) Transcriptional profiling of mouse microglia revealed the prostaglandin synthesis and regulation pathway to be significantly dysregulated during inflammation. The pathway diagram derived from differential microglial gene expression during LPS stimulation shows significantly up-regulated genes (red) and down-regulated genes (green). Importantly, the microglial EP2 receptor is significantly up-regulated (22.7-fold) compared with untreated microglia. The bilaminar culture system was used to assess microglia–macrophage communication on gene expression in adult mouse and human microglia and macrophages. (**b**) Adult mouse microglial mRNA expression of EP1 and 4 receptors treated with LPS (100 ng/mL) in the presence or absence of macrophages (Mϕ). (**c**) Adult mouse macrophage mRNA expression of EP1 and 4 receptors treated with LPS (100 ng/mL) in the presence or absence of microglia (Mg). (**d**) Adult human macrophage mRNA expression of EP2 treated with LPS (100 ng/mL) in the presence or absence of human microglia (Mg). (**e**) Adult mouse BMDM gene expression of four key inflammatory cytokines (IL-1β, TNF, IL-6, and IL-10) treated with LPS (100 ng/mL) in the presence or absence of EP2 agonist, Butaprost (1 μM). Statistical analysis; two-way ANOVA with Bonferroni corrections (*n =* 4–6), mean ± SEM. *p < 0.05; **p < 0.01; ***p < 0.001. Corresponding raw data (**S1 Data**). BMDM, bone marrow–derived macrophage; Mg, microglia.
(TIF)

S4 Fig Macrophages influence microglial cell death but not proliferation or process extension toward lesion. **(a)** Microglia viability was assessed by FACS in the coculture system using eflouro780 viability dye. Viability of microglia was not reduced significantly by BMDMs or LPS treatments alone. However, a combination of BMDMs and LPS caused a reduction of viable microglia compared with microglia alone. (**b**) Representative images of microglial proliferation in the presence or absence of BMDMs (Mϕ) and LPS. Cells were cocultured for 24 hours after the addition of Click-iT EdU to measure proliferation. (**c**) Quantification of Click-iT EdU colocalization with CD11b^+ve^ and DAPI-labeled nuclei shows an inflammatory stimulus (LPS) reduces microglial proliferation. BMDMs have no effect on proliferation of microglia in the presence or absence of LPS. (**d-g**) Proliferation of microglia in the presence or absence of macrophages in vivo after SCI. (**d**) Influx of LysM-eGFP+ myeloid cells at the epicenter of the injury after vehicle or clodronate treatment five days after SCI. (**e**) Quantification of total number of LysM-eGFP+ cells in spinal cord cross sections at the lesion epicenter. (**f**) Representative images of proliferation marker Ki67+ cells at the epicenter in the control and clodronate conditions. Arrow indicates Ki67+ microglial cells (LysM-eGFP^−ve^/ CD11b+). (**g**) Quantification of EGFP^−ve^/CD11b^+ve^ microglia colocalized with Ki67. There was no significant difference in the percentage of proliferating microglia between the two conditions. Scale bars = 100 μm. Statistical analysis: (**a-c**) two-way ANOVA with Bonferroni corrections (*n =* 3–4); (**e, g**) Student *t* tests (*n* = 3–4). Mean ± SEM. *p < 0.05; **p < 0.01; ***p < 0.001. (**h**) Live imaging of OHSCs was used to investigate initial microglial process extension toward laser lesions. OHSCs from Cx3cr1^+/gfp^ mice underwent two-photon (2P) laser lesion, and microglial process extension was tracked using ImageJ plug-in Mtrack over 15 minutes (colored lines indicate tracking of individual microglial processes over time). (**i-m**) Parameters measured after 2P laser lesion of OHSCs in five experimental conditions: (1) control slices, (2) OHSCs incubated with a feeder layer of BMDMs (Mϕ feeder), (3) OHSCs incubated with a feeder layer of BMDMs pre-stimulated with LPS (100 ng/mL) (Stim Mϕ feeder), (4) OHSCs incubated with BMDMs added directly onto the slice (5×10^5^/slice) (Mϕ on slice), (5) OHSCs incubated with LPS pre-stimulated (100 ng/mL, two hours) BMDMs added directly onto the slice (5×10^5^/slice) (Stim Mϕ on slice). No differences were seen in any of the parameters analyzed between different experimental conditions. (**n, o**) An in vivo model of TBI. (**n**) Representative live 2P images two hours after pressure-induced TBI between vehicle and EP2 receptor agonist (Butaprost; 1 μM)–treated mice. (**o**) Quantification of the number of “jellyfish” microglia after the two-hour imaging period. “Jellyfish” microglia represent the normal initial morphological response to pressure-induced TBI. This response was unchanged with local treatment (infused through thinned skull) of Butaprost. Statistical analysis: (**i-m**) one-way ANOVA with Bonferroni corrections (*n =* 3–4), (**o**) Student *t* test (*n =* 6). (**p**) Neutrophil infiltration and (**q**) CD86 expression in microglia were assessed by flow cytometry at three and seven days, respectively, after SCI in WT and CCR2 KO mice. Statistical analysis: Student *t* tests (*n* = 3–4). Corresponding raw data (**S1 Data**). EdU, 5-ethynyl-2′-deoxyuridine; FACS, fluorescently activated cell sorting; OHSC, organotypic hippocampal slice culture; SCI, spinal cord injury; TBI, traumatic brain injury; WT, wild-type; 2P, two-photon.
(TIF)

S5 Fig Flow cytometry gating information. Figure presents full gating strategies for Figs 1F, 4A, 5A and 6A.
(TIF)

S1 Data Raw data files. All relevant raw data for main and supporting figures.
(XLSX)

## Abbreviations

BMDM: bone marrow–derived macrophage
BMS: Basso Mouse Scale
CCR2: C–C chemokine receptor type 2
CNS: central nervous system
EdU: 5-ethynyl-2′-deoxyuridine
FACS: fluorescently activated cell sorting
HPGD: hydroxyprostaglandin dehydrogenase
IL: interleukin
IPA: ingenuity pathway analysis
LPS: lipopolysaccharide
MDM: monocyte-derived macrophage
MFI: mean fluorescent intensity
mPGES: microsomal prostaglandin E synthase-1
NF: nuclear factor
OHSC: organotypic hippocampal slice culture
PGE_2_: prostaglandin E_2_
RT-qPCR: quantitative real-time polymerase chain reaction
SCI: spinal cord injury
TBI: traumatic brain injury
TGF: Transforming growth factor
Treg: T-regulatory cell
WT: wild-type
