# Supplementary figures and images for: Peripherally derived macrophages modulate microglial function to reduce inflammation after CNS injury
Source: PLoS Biol. 2018 Oct 17;16(10):e2005264. doi: 10.1371/journal.pbio.2005264 (PMC6205650; doi:10.1371/journal.pbio.2005264)

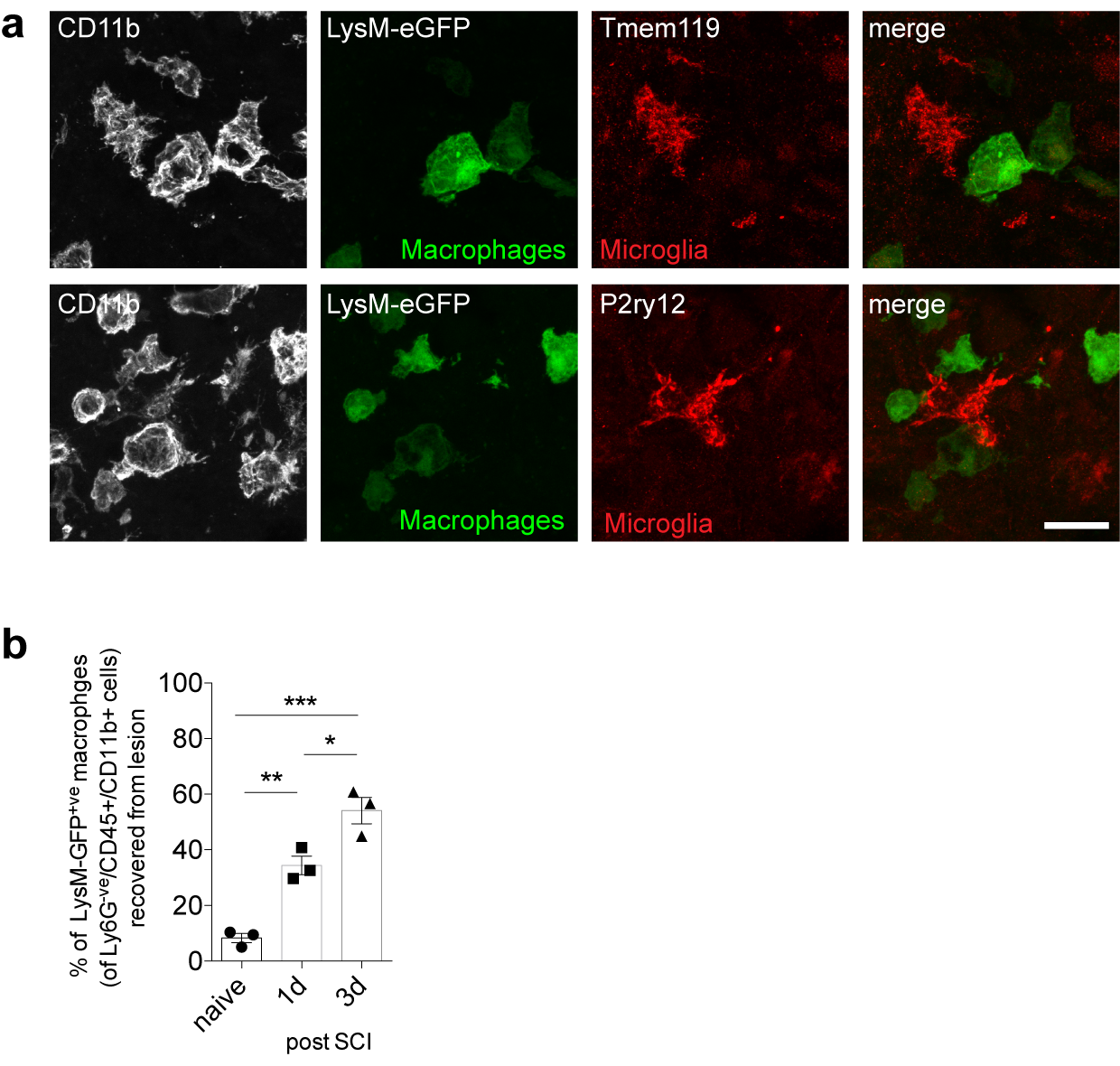

Supplement: S1 Fig — (a) Confocal z-stack images of infiltrating macrophages (green) (CD11b+ve/LysM-eGFP+ve/Tmem119−ve; top panel) and (CD11b+ve/LysM-eGFP+ve/P2ry12−ve; bottom panel) in position to interact with Tmem119+ve or P2ry12+ve microglial cells (red). Scale bar = 20 μm. (b) Increasing numbers of MDMs in the injured spinal cord after SCI. Myeloid cells were isolated from the uninjured or injured spinal cord of LysM-eGFP mice and LysM-eGFP+ve (macrophages) were quantified as a percentage of total CD45+/Ly6G−ve/CD11b+ cells. Statistics: One-way ANOVA with Bonferroni corrections (n = 3). Mean ± SEM. **p < 0.01; ***p < 0.001. Corresponding raw data (S1 Data). CNS, central nervous system; MDM, monocyte-derived macrophage; SCI, spinal cord injury. (TIF) [file pbio.2005264.s001.tif]

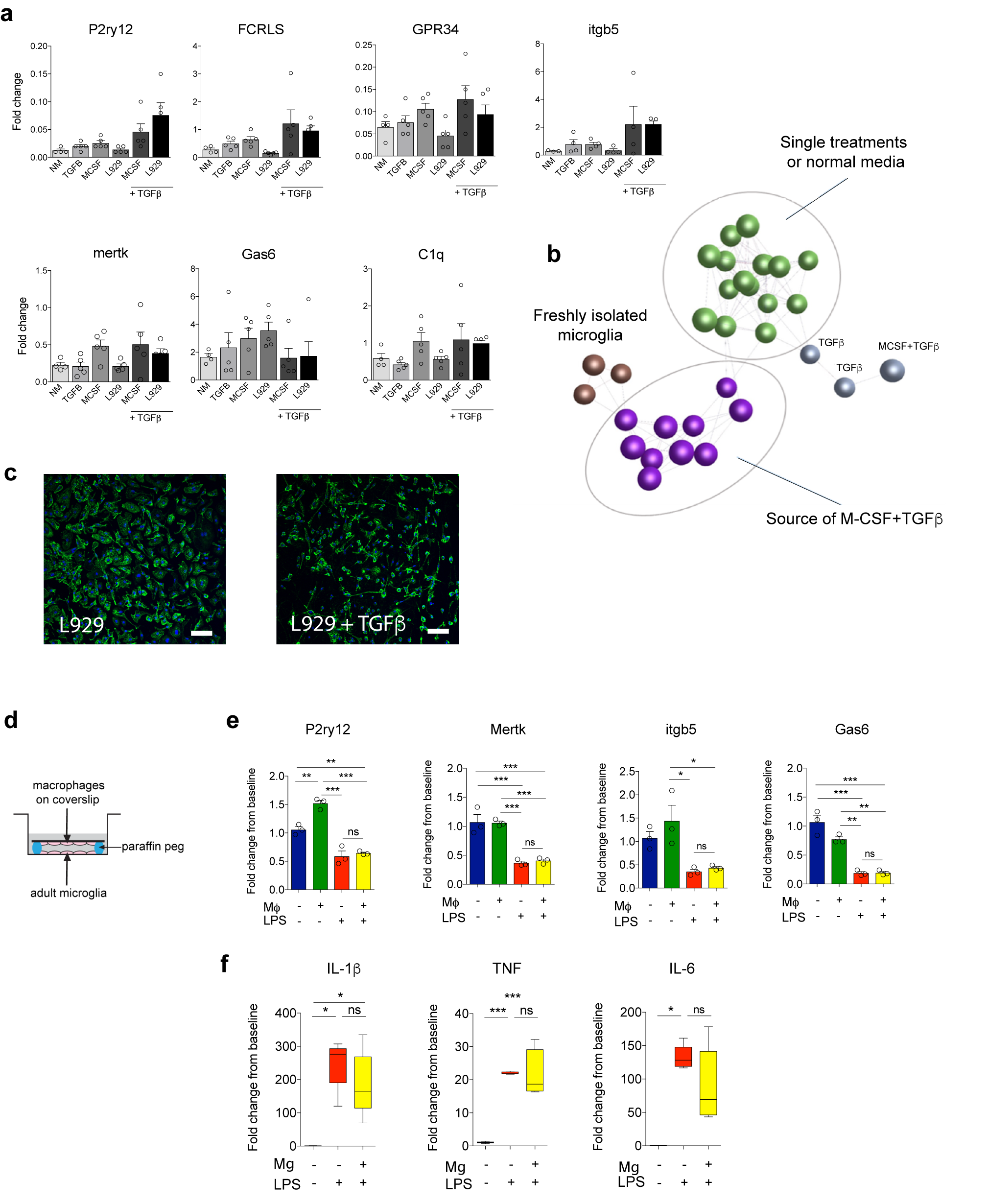

Supplement: S2 Fig — Primary adult mouse microglia were cultured under specific conditions in order to retain a transcriptional profile similar to in vivo adult microglia [11]. (a) Gene expression of seven microglia “signature” genes [11] in microglial cells cultured for seven days in DMEM-F12 media containing FBS (10%) (NM), with combinations of recombinant M-CSF (10 ng/mL), conditioned media from L-929 cells (rich in M-CSF; 10%) and recombinant human TGF-β1 (50 ng/mL). Fold changes are expressed relative to freshly isolated adult microglia. (b) Network graph showing sample-to-sample correlation of microglial signature gene expression shown in (a); analysis performed in Miru (Pearson correlation threshold, r ≥ 0.85). Nodes represent individual samples and edges the degree of correlation between them. The network graph was clustered using a Markov clustering algorithm, and samples were assigned a color according to cluster membership. Gene expression in freshly isolated microglia (brown) is most closely correlated with cultured microglia that had been treated with a source of M-CSF and TGF-β1 (purple). (c) Representative images of adult microglial cultures at seven days in the presence of L-929 conditioned media without and with TGF-β. (d) Schematic showing the bilaminar cultures. BMDMs were plated on coverslips on which small paraffin pegs were placed. These coverslips were then placed into wells containing adult microglia such that the two cell types were separated. (e) Adult mouse microglial gene expression of four microglia “signature” genes treated with LPS (100 ng/mL) in the presence or absence of macrophages (Mϕ). (f) Adult mouse microglia cultured with or without adult microglia and stimulated with LPS (100 ng/mL) show no difference in mRNA expression of IL-1β, TNF, and IL-6. Expression in microglia cultured alone is also shown. Statistical analysis; two-way ANOVA with Bonferroni corrections (n = 3–6), mean ± SEM. *p < 0.05; **p < 0.01; ***p < 0.001. Corresponding raw data (S1 D [file pbio.2005264.s002.tif]

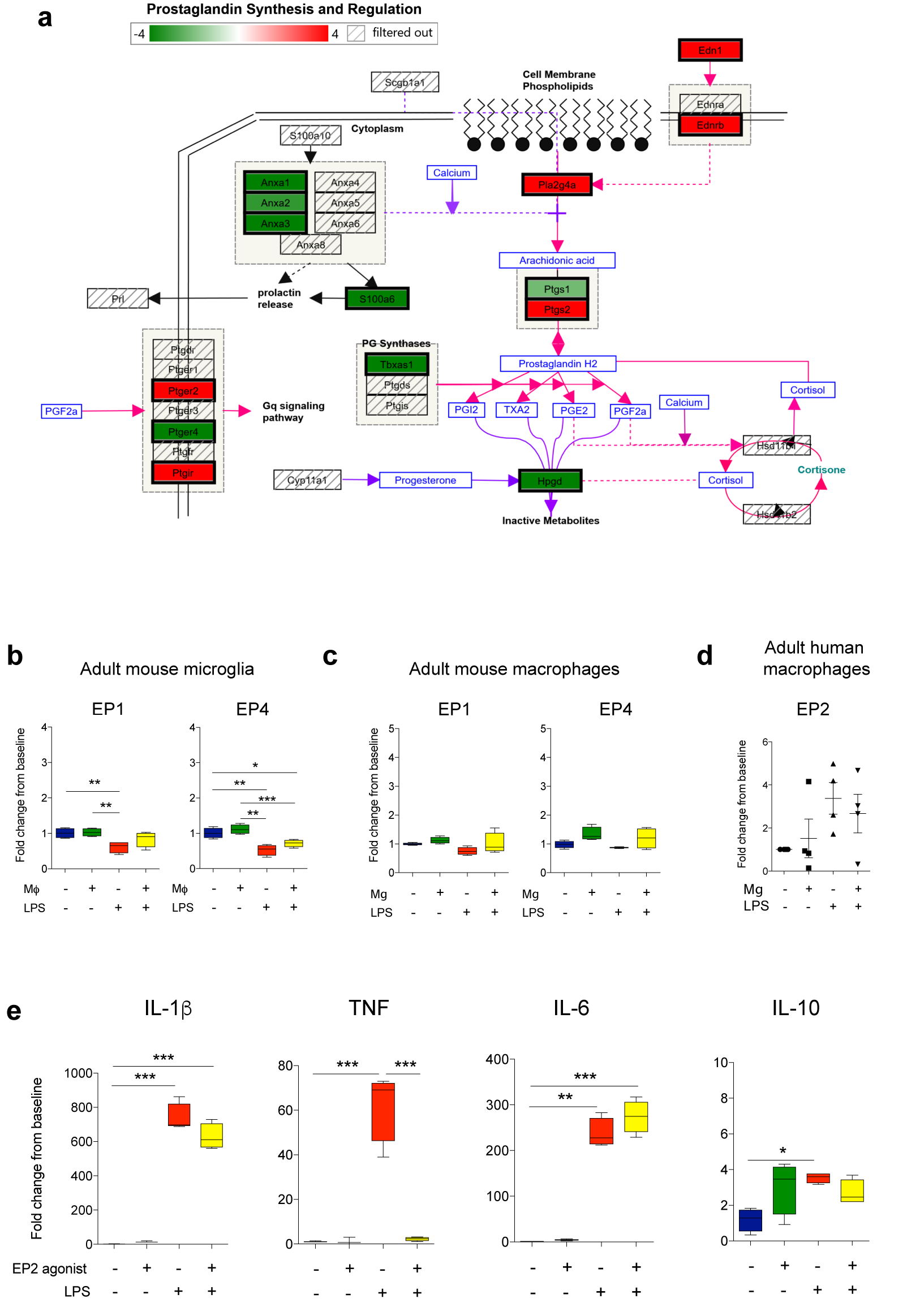

Supplement: S3 Fig — (a) Transcriptional profiling of mouse microglia revealed the prostaglandin synthesis and regulation pathway to be significantly dysregulated during inflammation. The pathway diagram derived from differential microglial gene expression during LPS stimulation shows significantly up-regulated genes (red) and down-regulated genes (green). Importantly, the microglial EP2 receptor is significantly up-regulated (22.7-fold) compared with untreated microglia. The bilaminar culture system was used to assess microglia–macrophage communication on gene expression in adult mouse and human microglia and macrophages. (b) Adult mouse microglial mRNA expression of EP1 and 4 receptors treated with LPS (100 ng/mL) in the presence or absence of macrophages (Mϕ). (c) Adult mouse macrophage mRNA expression of EP1 and 4 receptors treated with LPS (100 ng/mL) in the presence or absence of microglia (Mg). (d) Adult human macrophage mRNA expression of EP2 treated with LPS (100 ng/mL) in the presence or absence of human microglia (Mg). (e) Adult mouse BMDM gene expression of four key inflammatory cytokines (IL-1β, TNF, IL-6, and IL-10) treated with LPS (100 ng/mL) in the presence or absence of EP2 agonist, Butaprost (1 μM). Statistical analysis; two-way ANOVA with Bonferroni corrections (n = 4–6), mean ± SEM. *p < 0.05; **p < 0.01; ***p < 0.001. Corresponding raw data (S1 Data). BMDM, bone marrow–derived macrophage; Mg, microglia. (TIF) [file pbio.2005264.s003.tif]

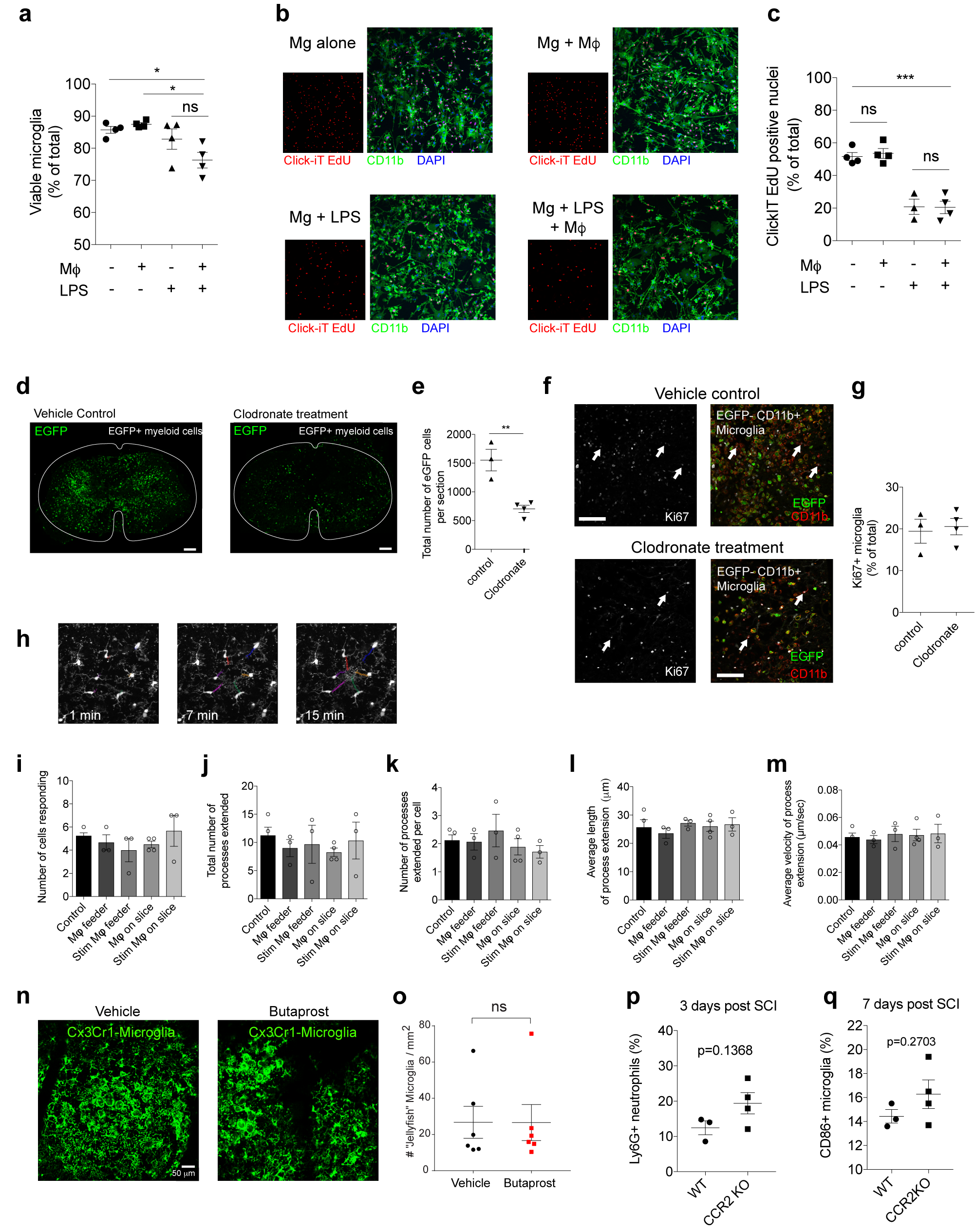

Supplement: S4 Fig — (a) Microglia viability was assessed by FACS in the coculture system using eflouro780 viability dye. Viability of microglia was not reduced significantly by BMDMs or LPS treatments alone. However, a combination of BMDMs and LPS caused a reduction of viable microglia compared with microglia alone. (b) Representative images of microglial proliferation in the presence or absence of BMDMs (Mϕ) and LPS. Cells were cocultured for 24 hours after the addition of Click-iT EdU to measure proliferation. (c) Quantification of Click-iT EdU colocalization with CD11b+ve and DAPI-labeled nuclei shows an inflammatory stimulus (LPS) reduces microglial proliferation. BMDMs have no effect on proliferation of microglia in the presence or absence of LPS. (d-g) Proliferation of microglia in the presence or absence of macrophages in vivo after SCI. (d) Influx of LysM-eGFP+ myeloid cells at the epicenter of the injury after vehicle or clodronate treatment five days after SCI. (e) Quantification of total number of LysM-eGFP+ cells in spinal cord cross sections at the lesion epicenter. (f) Representative images of proliferation marker Ki67+ cells at the epicenter in the control and clodronate conditions. Arrow indicates Ki67+ microglial cells (LysM-eGFP−ve/ CD11b+). (g) Quantification of EGFP−ve/CD11b+ve microglia colocalized with Ki67. There was no significant difference in the percentage of proliferating microglia between the two conditions. Scale bars = 100 μm. Statistical analysis: (a-c) two-way ANOVA with Bonferroni corrections (n = 3–4); (e, g) Student t tests (n = 3–4). Mean ± SEM. *p < 0.05; **p < 0.01; ***p < 0.001. (h) Live imaging of OHSCs was used to investigate initial microglial process extension toward laser lesions. OHSCs from Cx3cr1+/gfp mice underwent two-photon (2P) laser lesion, and microglial process extension was tracked using ImageJ plug-in Mtrack over 15 minutes (colored lines indicate tracking of individual microglial processes over time). (i-m) Parameters measured af [file pbio.2005264.s004.tif]

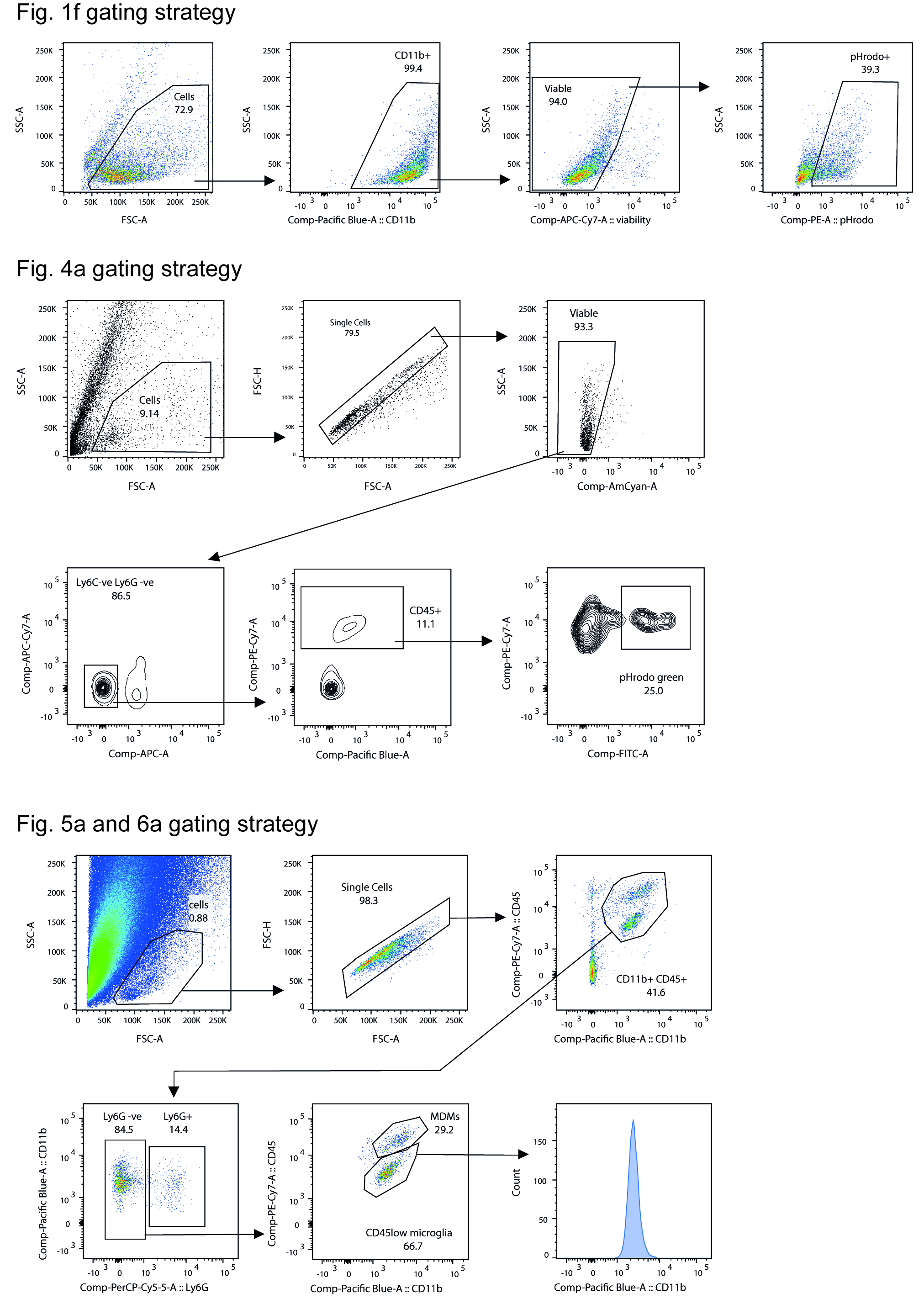

Supplement: S5 Fig — Figure presents full gating strategies for Figs 1F, 4A, 5A and 6A. (TIF) [file pbio.2005264.s005.tif]
